# Influence of Glucose Availability and CRP Acetylation on the Genome-Wide Transcriptional Response of *Escherichia coli*: Assessment by an Optimized Factorial Microarray Analysis

**DOI:** 10.3389/fmicb.2018.00941

**Published:** 2018-05-23

**Authors:** Daniel V. Guebel, Néstor V. Torres

**Affiliations:** ^1^Biotechnology Counselling Services, Buenos Aires, Argentina; ^2^Systems Biology and Mathematical Modelling Group, Department of Biochemistry, Microbiology, Cellular Biology and Genetics, Institute of Biomedical Technologies, Center for Biomedical Research of the Canary Islands, University of La Laguna, San Cristóbal de La Laguna, Spain

**Keywords:** *Escherichia coli*, post-translational modification, lysine acetylation regulation, CRP, acetyl transferase PatZ, deacetylase CobB, glucose, factorial analysis

## Abstract

**Background:** While in eukaryotes acetylation/deacetylation regulation exerts multiple pleiotropic effects, in *Escherichia coli* it seems to be more limited and less known. Hence, we aimed to progress in the characterization of this regulation by dealing with three convergent aspects: the effector enzymes involved, the master regulator CRP, and the dependence on glucose availability.

**Methods:** The transcriptional response of *E. coli* BW25113 was analyzed across 14 relevant scenarios. These conditions arise when the wild type and four isogenic mutants (defective in deacetylase CobB, defective in N(ε)-lysine acetyl transferase PatZ, Q- and R-type mutants of protein CRP) are studied under three levels of glucose availability (glucose-limited chemostat and glucose-excess or glucose-exhausted in batch culture). The Q-type emulates a permanent stage of CRP^acetylated^, whereas the R-type emulates a permanent stage of CRP^deacetylated^. The data were analyzed by an optimized factorial microarray method (Q-GDEMAR).

**Results:** (a) By analyzing one mutant against the other, we were able to unravel the true genes that participate in the interaction between Δ*cobB*/Δ*pat*Z mutations and glucose availability; (b) Increasing stages of glucose limitation appear to be associated with the up-regulation of specific sets of target genes rather than with the loss of genes present when glucose is in excess; (c) Both CRP^deacetylated^ and CRP^acetylated^ produce extensive changes in specific subsets of genes, but their number and identity depend on the glucose availability; (d) In other sub-sets of genes, the transcriptional effect of CRP seems to be independent of its acetylation or deacetylation; (e) Some specific ontology functions can be associated with each of the different sets of genes detected herein.

**Conclusions:** CRP cannot be thought of only as an effector of catabolite repression, because it acts along all the glucose conditions tested (excess, limited, and exhausted), exerting both positive and negative effects through different sets of genes. Acetylation of CRP does not seem to be a binary form of regulation, as there is not a univocal relationship between its activation/inhibitory effect and its acetylation/deacetylation stage. All the combinatorial possibilities are observed. During the exponential growth phase, CRP also exerts a very significant transcriptional effect, mainly on flagellar assembly and chemotaxis (FDR = 7.2 × 10^−44^).

## Introduction

It is well known that microbial cells continuously sense their external and internal environment, with this information being filtered and integrated to trigger responses that allow them to best adapt to these stimuli (Snitkin and Segrè, 2008; Zhao et al., 2016). Beyond the “short-term adaptations” provided by the allosteric regulations working alongside the extensive set of protein post-translational modifications, changes in the transcriptional responses are the main mechanisms for achieving the “long-term adjustments” required (Shimizu, 2014). So, there are good reasons to analyse changes in the global transcriptome of microorganisms. To this end, one technique that is widely used is the high-density array of oligonucleotides (microarrays).

In fact, microarrays enable the analysis of the complete *Escherichia coli* transcriptome by monitoring the labeled hybridization of around ten thousand probes. Moreover, transcriptome analysis also provides information about the non-coding RNAs (anti-sense RNAs and microRNAs), which have increasing importance as additional layer of regulation at post-transcriptional level, not only in eukaryotes but also in bacteria (Delihas, 2015; Cech et al., 2016; Tronnet et al., 2016).

Here, we will focus on the analysis of changes in the transcriptome of some *E. coli* strains (wild-type and isogenic mutants) when these bacteria are challenged with different levels of a typical carbon source such as glucose. The analyses include three well-defined scenarios: the case of unrestricted glucose availability (exponential phase of batch culture), the case of glucose deprivation (stationary phase of batch culture), and the stage of transition between these extremes (glucose-limited chemostat culture). Of course, it must be noted that multiple experimental studies of this type have been done over the last 10 years (see Table 1). Also, some reviews are available (Fic et al., 2009; Hu et al., 2010; Bernal et al., 2014; Choudhary et al., 2014; Drazic et al., 2016; Wolfe, 2016). What then justifies the present study?

**Table 1:** Some experimental studies previously done with the aim of analyzing at the genome-wide level the effect of glucose availability and/or catabolite repression by the CRP protein.

**Study**	***E. coli* strain**	**Experimental system**	**Microarray**	**Commentary**
Weber et al., 2005	K12 MC41000 derivative	Batch, (LB and M9 culture medium)	Built in home Criteria: |Fold-change|≥2 and *t*-test with *p* ≤ 0.05	Apply different types of stresses. Define the general stress response
Franchini and Egli, 2006	K12 MG1655	Batch and glucose-limited chemostat (D = 0.3 h^−1^)	MWG-Biotech AG Criteria: *t*-test > 2 or *t*-test < 0.5 with *p* ≤ 0.05	Consider both short-term steady state (40 h) and long-term steady state (500 h) in the chemostat
Lemuth et al., 2008	K12 W3110 (DSM5911)	Fed-Batch with constant feeding of glucose (D < 0.16 h^−1^)	OligoSet (MGW, Germany) Criteria: LIMMA with FDR ≤ 0.05	Compare transcription under progressive glucose starving against the one in exponential growth phase
Khankal et al., 2009	W3110 (ATCC 27325)	Batch (LB and LB plus glucose)	Genechip *E. coli* Genome 2 (Affymetrix) Criteria: LIMMA with FDR < 0.05	Compare transcription between wild type and mutants *crp* with different sensitivity to c-AMP for activation under four culture conditions
Nahku et al., 2010	K12 MG1655	Accelerostat (chemically defined culture medium) (D ranged between 0.3 and 0.47 h^.−1^ with acceleration factor 0.01 h^−2^)	*E. coli* Genome Oligo Set V1 (Operon Biotechnol Inc.) Use KTH package for data processing (GSE 18183)	Transcriptome D = 0.3 h^−1^ and D = 0.42 h^−1^. Monitoring the production of acetic acid together with other fermentative parameters
Yao et al., 2011	*E. coli* BW25113	Chemostat (D = 0.2–0.7 h^−1^)	RT-PCR	*crp, cra, mlc*, and *rpoS* decreased with μ, while f*adR, iclR, soxR, soxS* increased. Study several mutants
Borirak et al., 2014	K12 MG1655	Chemostat plus pulse of glucose in M9 medium	RT-PCR	Analyse physiological parameters after the pulse on the steady state of culture with the wild-type strain
Borirak et al., 2015	K12 MG1655	Chemostat plus pulse of glucose in M9 medium	G4813A-020097 (Agilent) + 311 probes e-Array (Agilent) + Proteomics Quantification method developed by the authors for analysis of temporal series (Array Express: E-MTAB-2398)	The transient transcriptional response after glucose pulse (*t* = 0, 5, 15, 30, 60 min) on the steady state of culture with the wild-type strain is analyzed
Franchini et al., 2015	K12 MG1655	Glucose-limited chemostat and LB modified (D = 0.3 h^−1^)	MWG-Biotech AG	Defective mutants Δ*rpoS*, Δ*crp*, Δ*cya*
Castaño-Cerezo et al., 2014	K12 BW25113	Batch and glucose-limited chemostat (D = 0.23 h^−1^)	Gene Chip *E. coli* Genome 2 (Affymetrix) (FDR ≤ 0.05)	Defective mutants Δ*cobB* (deacetylase) and Δ*patZ* (acetyl-transferase)
Peebo et al., 2015	K12 (BW25113)	Accelerostat		
Vital et al., 2015	K12 (MG1655) IA-1 TW11588 –Clade IV TW109308-Clade V	Batch and chemostat (chemical defined medium)	RNAseq	Analyse four natural strains from different origins, their sequencing, and regulatory properties

Inspection of Table 1 reveals some important aspects that justify the need to perform the present study. Hence, only a few studies simultaneously integrate the three biological scenarios employed herein. Moreover, our study aims not only to analyse the transcriptional effect of the glucose availability, but also to establish the extent to which these effects are specifically regulated through acetylation/deacetylation of the CRP protein. In fact, this protein is the main protein responsible for catabolite repression (Deutscher, 2008). Together with RcsB and RpoB, CRP is one of the three main transcriptional regulators known in *E. coli* as influenced by acetylation (Zhang et al., 2009).

There are not many previous studies addressing catabolite repression from the point of view of its regulation by acetylation/deacetylation of CRP. Often, when studies referred to regulation by acetylation, they were not actually addressing the problem of glucose availability (Lima et al., 2011; Ma and Wood, 2011; Weinert et al., 2013a; Zhang et al., 2013; Baeza et al., 2014). Moreover, the mechanism of lysine-acetylation/deacetylation is also present in different proteins of *E. coli* as a type of post-translational modification (Ma and Wood, 2011; Liu et al., 2014; Castaño-Cerezo et al., 2015; Fraiberg et al., 2015; Drazic et al., 2016).

Although most of the studies in Table 1 used microarrays, it is well known that this tool has major limitations (Guarnaccia et al., 2014; Yang et al., 2014; Chrominski and Tkacz, 2015). Many of these limitations derive from the current algorithms applied, such as the Empirical Bayes and Benjamini-Hochberg algorithms. To avoid these problems, we have developed an alternative algorithm called Q-GDEMAR (**Q**uantile-**G**aussian **de**convolution of **m**icro**ar**rays), which provides a better sensitivity of detection together with a low false discovery rate (FDR) (Guebel et al., 2016).

Further, all the studies in Table 1 only considered pair-wise comparisons. The microarray data in our analyses, however, will be arranged as a factorial design, thus allowing us to account for the interaction between the variables. Furthermore, unlike the studies shown in Table 1 that “summarized” their microarrays (Irizarry et al., 2003), to optimize our study, the microarray data will be analyzed here at the probe-level. In any case, of special importance is the fact that all the technical variants that we will use for the microarray analyses in the present investigation have recently been discussed and tested successfully (Guebel and Torres, 2016; Guebel et al., 2016).

Note in Table 1 the occurrence of additional sources of variability, which affect the comparability of results among the studies. Thus, due to the different strains used by different authors, significantly different regulatory features can be expected, even though all the strains assayed belong to the *E. coli* taxon (Yoon et al., 2012; Castaño-Cerezo et al., 2015; Vital et al., 2015; Monk et al., 2016). Moreover, the presence of different trademarks in the microarray studies in Table 1 might imply a higher prevalence of false responses (cross-reactivity) in some cases due to the shorter length of probes in some kits (Chou et al., 2004). Importantly, the interpretations are conditioned not only by the type of computing algorithm used, but also by the application of different cut-off criteria (for example, see in Table 1 the use of *t*-student arbitrary values together with non-adjusted *p* values for multi-comparisons; *p* adjusted by Bonferroni; *p* adjusted by FDR). Taken together, these factors inexorably lead to the identification of different sets of differential genes and hence produce different biological inferences.

In brief, herein we advance in the characterization of regulatory acetylation mechanisms exerted by the DNA-binding dual regulator CRP (CRP: cAMP receptor protein) and their relation with glucose availability. To this end, we re-analyse two sets of previously published data, but unlike in the original reports, herein the transcriptional responses are crossed with the different conditions of glucose availability, and the microarray data are dealt with using our optimized post-processing approach by factorial Q-GDEMAR. In addition, the criteria applied to consider the control conditions are also reformulated.

## Materials and methods

### Strains and conditions tested

Two sources of microarray data are re-analyzed herein. The first set concerns the global transcriptional responses to defective acetylation-deacetylation *E. coli* mutants. This data set has been gathered by Castaño-Cerezo et al. (2014), and was retrieved for our study from the GEO database (access code: GSE62094). The second set concerns the overall transcriptional responses of *E. coli* to mutations in the *crp* gene, which codifies the DNA-binding dual regulator CRP. This information has been gathered by Écija-Conesa and de Diego (2007) (GEO database, access code: 96955)^1^ and published latter (Davis et al., 2018). These set of data are highly consistent since they came from the same laboratory, uses the same host *E. coli* strain, culture media, operational conditions, and analytics. Moreover, the analyses of both data sets are independent one of the other.

In the first case, the analysis encompasses the comparison of transcriptional responses to six experimental conditions. These scenarios result from testing three variants of the *E. coli* BW25113 strain (wild type, Δ*patZ*, Δ*cobB*) when cultured under two modes of bioreactor operation (batch and chemostat). The Δ*cobB* mutant refers to a strain defective in the c*obB* gene (ycfY), which encodes the protein-lysine deacetylase and desuccinylase enzyme (Uniprot: P75960). This enzyme belongs to the family of sirtuins and is NAD^+^-dependent. By contrast, the mutant Δ*pat*Z refers to a strain defective in the *patZ* gene (yfiQ), which encodes the peptidyl-lysine acetyl-transferase enzyme (Uniprot: P76594). The samples coming from the exponential phase of the batch (i.e., culture without glucose limitation) correspond to a specific growth rate μ_max_ = 0.62 h^−1^. The samples coming from the chemostat (glucose-limited culture) correspond to a Dilution Rate = 0.23 h^−1^ and were taken at the steady state achieved after five residence times.

In the second case, the analysis encompasses the comparison of the transcriptional responses to eight experimental conditions. These scenarios result from testing four other *E. coli* BW25113 genetic variants [wild type; ΔN (a cloning control); Δ*crp*(R-mutant), Δ*crp* (Q-mutant)] under two stages of batch culture (exponential and stationary phase). In fact, in one of these mutants, the strain *E. coli* BW25113 was engineered to produce a modified CRP protein in which the Lysine (K) 100, a critical residue for the CRP activity (Baeza et al., 2014), was substituted to codify an Arginine (R-mutant). In the second type of mutant, the K100 was substituted to codify a glutamine (Q-mutant). Importantly, the objective of the mutant Δ*crp*(R-type) is to emulate a constitutive stage of deacetylation in Lysine 100 of CRP, whereas the objective of the mutant Δ*crp*(Q-type) is to emulate a stage of constitutive acetylation of Lysine 100 in the same protein.

In this last series of experiments, an *E. coli* strain BW25113 defective in the *crp* gene was used as host of the cloning. The authors indicate that since they could not insert the modified genes at the native position, the variants of the *crp* gene (Q-type and R-type) were cloned on the *paaH* gene. This gene, which codes for 3-hydroxyadipyl-CoA dehydrogenase enzyme, is located within the *paa*ABCDEFGHIJK operon. So that the cloned variants could be transcribed independently on the hosting operon promoter, the gene variants were cloned together with the promoter corresponding to the native *crp* gene. Moreover, to account for the effects caused by the change in the position, a third type of mutant that works as cloning control, called ΔN, was considered. Thus, the “normal” *crp* gene and its associated promoter were cloned at the position of the *paaH* gene as well.

In both series of experiments, the bacterial cultures were done at 37°C, using minimal defined medium (MM9) with glucose 10 mM (pH = 7.4) and full aerobic conditions. However, while in the first series each condition was run in triplicate, in the second series each condition was run in duplicate. The samples corresponding to the exponential phase were taken at an optical density = 0.5, while the samples corresponding to the stationary phase samples were taken at an optical density = 1.8. For the microarray determinations, the authors indicate that RNA was extracted and purified from the culture samples, subjected to current quality control procedures, and finally run on Gene Chip *E. coli* Genome 2.0 (Affymetrix, USA) (for details see the original articles). Importantly, this microarray kit comprises 10,207 probes, from which only 8,662 have a gene assigned.

### Factorial microarray analysis

The log_2_-normalized data of the microarray are subjected herein to a post-processing elaboration by the Q-GDEMAR method (Guebel et al., 2016). This performs a computational deconvolution of the central region of the data distribution. The parametric characterization of this region in terms of a Gaussian distribution provides narrower limits to the genes whose expression fluctuates only stochastically. The comparison of these limits with the overall data distribution ultimately allows us to determine, with greater sensitivity and lower FDR, which probes are being differentially hybridized. The specific protocol for 2 × 2 factorial microarray analysis and the justification for why the probes are not subjected to median-polishing summarization have been described elsewhere (Guebel and Torres, 2016).

### Ontology analysis

This was performed by the software DAVID 6.8 (Huang et al., 2009; Jiao et al., 2012). This is a free web service (https://david.ncifcrf.gov/). We have used the default settings provided by the program, while contrasts were performed against the list corresponding to the whole genome of *E. coli* as background.

### Other sources of information

For reference pathways, maps were based on the KEGG database (available at http://www.kegg.jp/) (Kanehisa and Goto, 2000; Kanehisa et al., 2017). The information about *E. coli* genes was based on the EcoCyc database (available at https://ecocyc.org/) (Karp et al., 2014; Keseler et al., 2017).

## Results and discussion

### Substrate availability and genome-wide transcription

In previous analyses of Castaño-Cerezo et al. (2014), the focus was placed on the study of two mutant strains (Δ*cobB* and Δ*patZ*). There, the wild-type strain was considered only as the “control” against which the effects of the mutations were contrasted. However, besides assessing the performance of the mutants by using an optimized microarray post-processing, we also analyse herein the performance of the wild-type strain itself (see Table 2).

**Table 2:** Number of differentially expressed genes detected when the microarray data are analyzed by the Q-GDEMAR method according to the type of contrast assessed (culture mode and strain type).

**Experiment**	**A**	**B**	**C**	**D**
**Comparison (Strains)**	**Chemostat 1-Batch (WT vs. WT)**	**Batch-Batch (WT vs. WT)**	**Chemostat 2 (**Δ***cobB*** **vs. WT)**	**Chemostat 3 (**Δ***patZ*** **vs. WT)**
Ratio >>1	104 (1.9)	56 (2.8)	125 (0.9)	119 (3.6)
Ratio <<1	NDAS^*^	6 (0.0)	16 (2.8)	64 (4.1)

*(A) Chemostat 1-Batch: Ratio between the transcriptional response under glucose-limited conditions (chemostat, D = 0.23 h^–1^) and the response under glucose excess (batch exponential phase, μ_max_ = 0.62 h^–1^); (B) Batch-Batch: Ratio between the transcriptional response in the stationary phase and the response in the exponential phase, both from batch culture; (C) Chemostat 2: Ratio between the transcriptional response of ΔcobB mutant and the response of the wild-type strain (WT), both growing in glucose-limited chemostats (D = 0.23 h^–1^); (D) Chemostat 3: Ratio of transcriptional response of ΔpatZ mutant and the response of wild-type strain (WT), both growing in glucose-limited chemostats (D = 0.23 h^–1^). The values in parentheses indicate the associated false discovery rate (%FDR). For details see Tables S1–S4*.

* *NDAS, not detectable as significant*.

In Table 2, a ratio value significantly >1 means that genes corresponding to the condition considered at the numerator are up-regulated (or equivalently, that the genes corresponding to the condition considered at the denominator are down-regulated). Conversely, a ratio value significantly <1 means that genes corresponding to the condition considered at the denominator are up-regulated (or equivalently, that the genes corresponding to the condition considered at the numerator are down-regulated).

With these criteria of interpretation in mind, several aspects of Table 2 are noticeable. First, we note the small number of differentially expressed genes observed in each condition in spite of the high number of probes present in the microarray (*n* = 8,662). Second, we note the low values of FDR achieved throughout the analysis by the optimized microarray post-processing used herein. Note that the FDR values computed cover the entire set of associated genes (Guebel et al., 2016).

Third, most of the genes operating during the exponential growth phase (where the wild-type strain achieved a μ_max_ = 0.62 h^−1^) seem to continue being transcribed even during the stationary phase (where the growth of the strain has ceased). In fact, only six genes show a significant enhanced transcription during the exponential growth phase (second row, Experiment B), whilst 56 genes appear dysregulated during the stationary phase (first row, Experiment B). The small group of genes dysregulated during the exponential growth phase codify proteins involved in the metabolism of sulfur compounds such as methionine, cysteine, S-adenosyl methionine (*cysD, cysP, metE* genes), the novo biosynthesis of pyrimidines and glutamine metabolism (*pyrD, carA* genes), the biosynthesis of phenyl-alanine, tyrosine, and tryptophan (*aroG* gene).

**Figure 1:**
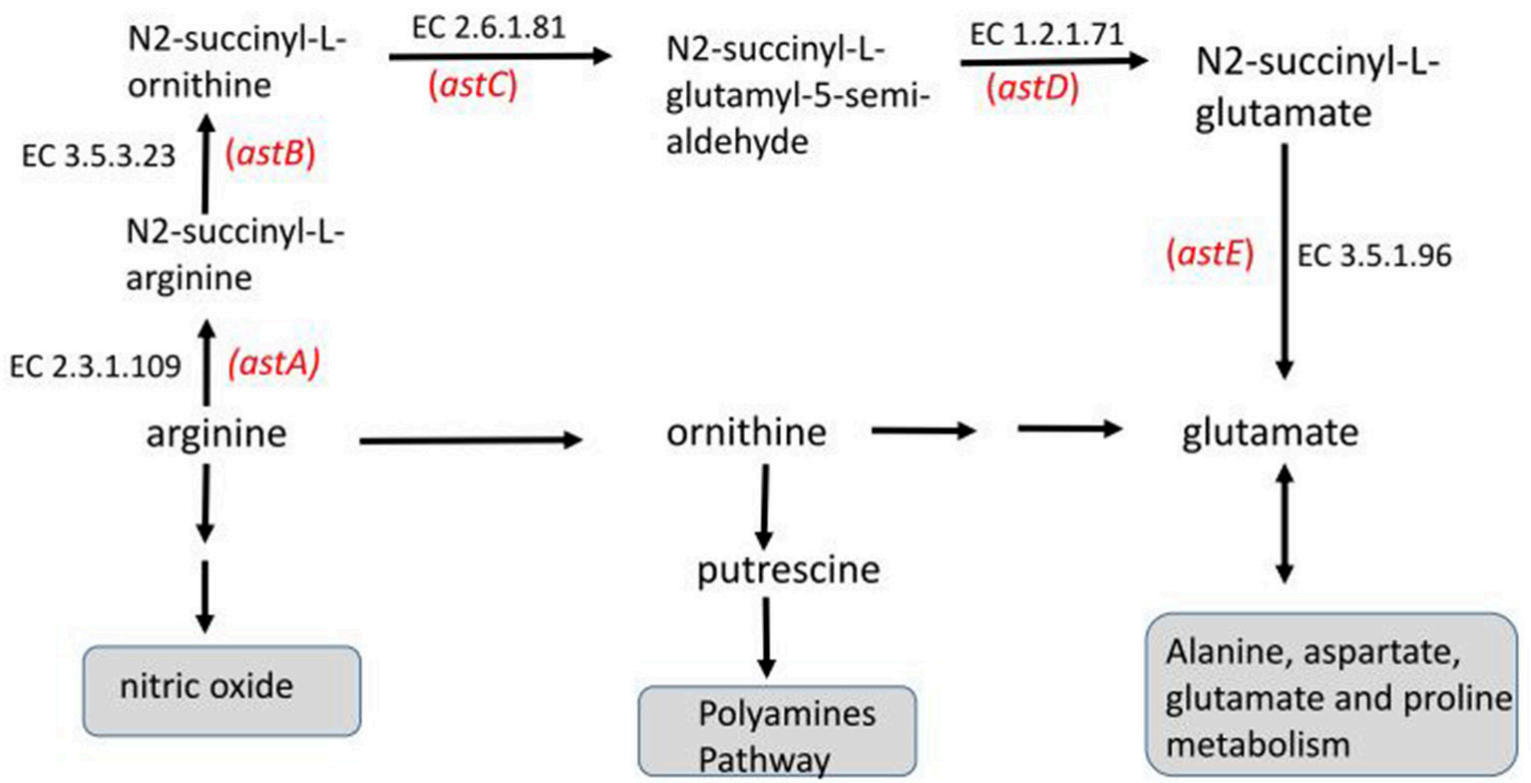
Arginine catabolic routes mapping the sub-set of five enzymes codified by the genes dysregulated in the microarray data corresponding to the stationary phase of batch culture with the wild type *E. coli* strain (first row, Experiment B, Table 2). The enzymes are indicated by their Enzyme Commission (EC) number, while the names of the genes that codify them are indicated in red. The diagram is a simplified adaptation based on KEGG Pathways (map 00330) after Ontology DAVID 6.8 software processing (FDR = 8 × 10^−4^).

Importantly, by the ontology analysis of the 56 differential genes detected for the stationary phase with the wild-type strain we identified as significant classes a set of four genes that codify periplasmic proteins with dodecin-like structure (ECs5165, ECs0884, ECs5164, ECs5175; FDR = 2.1 × 10^−2^) and another set of five genes which codify enzymes involved in L-arginine degradation (*astA* [arginine N-succinyltransferase], *astB* [N-succinylarginine dihydrolase], *astC* [bifunctional enzyme succinylornithine transaminase/N-acetylornithine transaminase], *astD* [N-succinylglutamate 5-semialdehyde], *astE* [succinylglutamate desuccinylase]; FDR = 8 × 10^−4^) (see Figure 1).

The biological role of the dodecin-like proteins is not well-known. However, based on their structural similarity with the dodecin molecule, these proteins could also have a protective role due to their capacity to sequester molecules containing oxidized flavin-moieties (Grininger et al., 2009). On the other hand, arginine, in addition to its structural role as amino acid, is also required as N donor for the biosynthesis of the messenger nitric oxide (NO). Moreover, through its transformation to ornithine, arginine is used for the biosynthesis of polyamines (putrescine and spermidine). Thus, is inferred that these two activities of arginine might be affected during the stationary phase.

Other groups of genes also appeared up-regulated during the stationary phase although they did not achieve the threshold of significance in the ontology analysis. In fact, we detected a group of five genes belonging to the *hya*ABCDE operon that codify the different subunits of the enzyme hydrogenase 1, two genes related with biofilm formation (*bssR*, and *bsmA*), several genes related with stress response (*gadC, ytfQ, csiE, ymjC, yjbJ*), and three genes related with the carbohydrates usage (*ara*F, *ytf* R, *sgcC*). Noticeably, around 50% of the total differential genes detected at the stationary phase correspond to hypothetical proteins (see Table S2).

Consistent with our claim about the persistence of most of the genes operating under the exponential phase in further culture phases, from Experiment A in Table 2 it can be seen that only 104 differential genes appear to be up-regulated due to the glucose limitation in “chemostat 1” (Dilution rate = μ = 0.23 h^−1^, a representative fixed point within the interval of the progressive deceleration phase in batch culture). As was previously commented, only 56 genes appear to be differentially up-regulated at the stationary phase, when the growth of the strain has ceased.

Despite that most of the genes operating at the exponential phase persist further, the sets of differential genes detected in the two analyzed transitions seem to be characteristic of each step. In fact, only 10 out of 160 total up-regulated genes (i.e., 6.2%) were common between the glucose-limited and the stationary phase. These common genes are the following: *c2666, yjfN*, c1304, *prpB*, c1608, *ycfQ, bsmA*/*yjfO, c5447, mocA*/*ygfJ, ydcS*. There, the gene *ydcS* codifies a protein with activity of polyhydroxybutyrate (PHB) synthase, the *bsmA*/*ycfO* gene is related to peroxide resistance, biofilm stress, and motility, while the *mocA*/*ygfJ* gene codifies the CTP:molybdopterin cytidylyltransferase enzyme.

**Figure 2:**
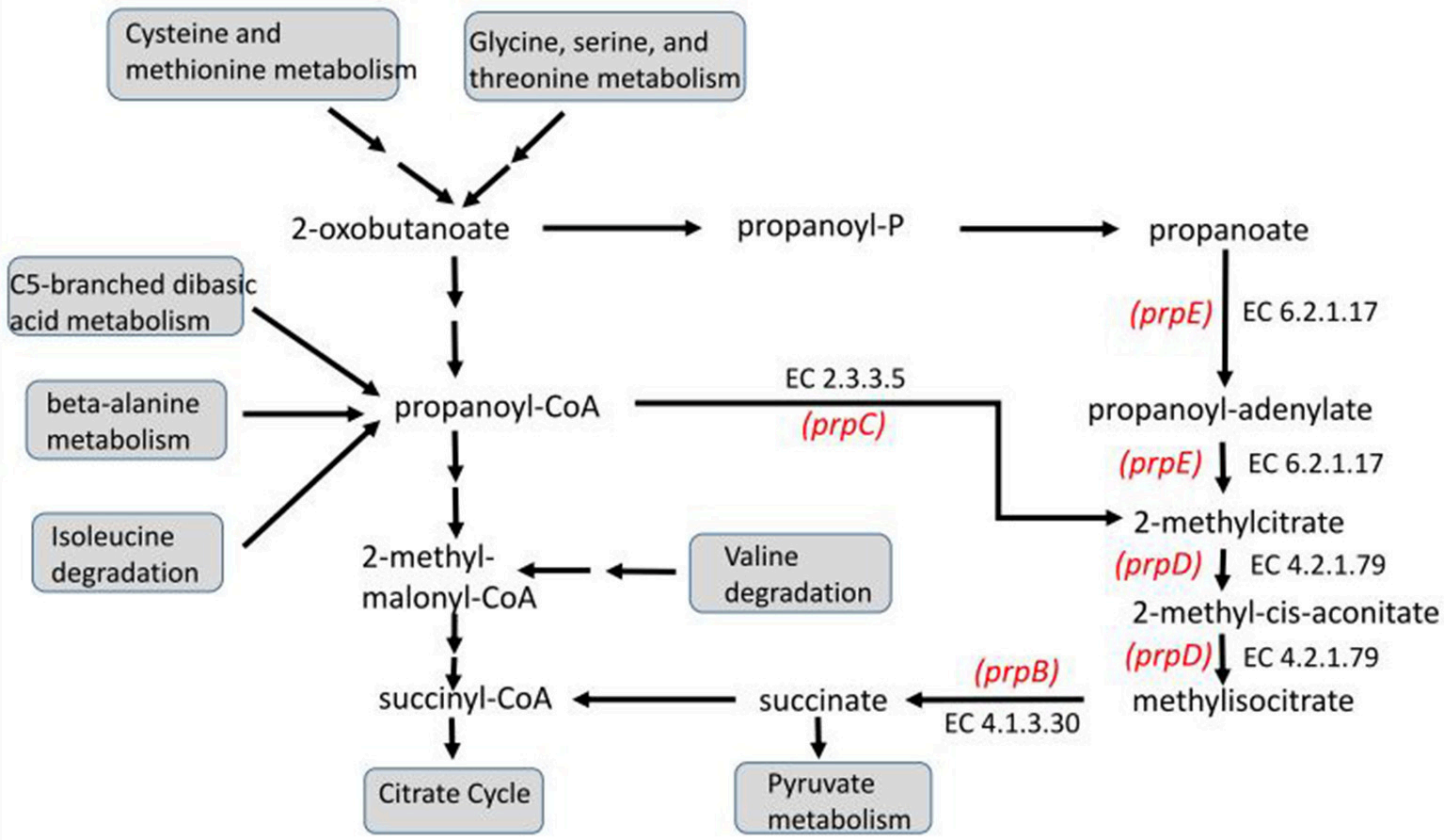
Propanoate metabolism and methylcitrate cycle mapping the sub-set of four enzymes codified by the genes dysregulated in the microarray data corresponding to the glucose-limited chemostat culture with the wild-type *E. coli* strain (first row, Experiment A, Table 2). The enzymes are indicated by their Enzyme Commission (EC) number, while the names of the genes that encode them are indicated in red. The diagram is a simplified adaptation based on KEGG Pathways (map 00640) after Ontology DAVID 6.8 software processing (FDR = 1 × 10^−5^).

Concerning the 104 genes detected in the wild type, the transcription of which increased as dependent on the glucose limitation (see Table S1), 48 genes codify proteins with no annotated functions (hypothetical proteins). Moreover, two genes correspond to non-coding RNA (*ryeA* and *cyaR*). The *cyaR* gene is a direct target of CRP and, by its negative effect on the OmpX translation, indirectly enhances the expression of fimbria proteins. The expression of *cyaR* also negatively influences the translation of the protein codified by the *nadE* gene, which is involved in the NAD salvage pathway. Importantly, on the remaining 56 differential genes detected, the ontology analysis indicates that the only significantly enriched term is the one corresponding to propanoate metabolism (FDR = 1 × 10^−5^). The genes involved in this pathway are induced by CRP and belong to the operon *prp*BCDE (*prpB* [methyl-isocitrate lyase], *prpC* [2-methylcitrate synthase], *prpD* [2-methylcitrate dehydratase], and *prpE* [propanoate-CoA ligase]). This finding is in agreement with the trend observed in another study that used RNA sequencing (Vital et al., 2015). Propanoate metabolism is related to the degradation routes of the amino acids cysteine, methionine, glycine, serine, threonine, and isoleucine (see Figure 2).

The fourth noticeable point is that the number of up-regulated genes in the so-called “chemostat1-batch” comparison, which is based exclusively on the wild-type strain (Experiment A, Table 2), is as high as the number detected when both mutant strains are compared against the wild-type strain (Experiments B and C, Table 2).

In brief, while the third point above raises the question about the nature of the transcriptional changes implied in the physiology of *E. coli* when working under increasingly glucose-limited conditions, the fourth point raises the question as to whether the inferences from the expression profile of mutants can really be attributed to the mutations carried out by the strains analyzed, or whether they must be attributed to other causes. In the following, we will present our exploration to determine the extent to which the two questions are separable or interdependent.

### Interaction between growth rate and transcription profile

Although the use of mutant strains in Experiments C and D pursued the objective of enhancing the visualization of the acetylation-deacetylation regulation in *E. coli*, the data from Table 2 show that the growth rate leads by itself to substantial changes in the transcriptome, even when no planned mutations were present. In other words, the effects of the mutations overlap with the effects of the growth rate. In statistical terms, it is said that the two variables are “confounded.” For this reason, and exploiting the implicit structure of a 2 × 2 factorial experimental design present in the data, we will proceed to determine rigorously which genes have an expression with significant dependence on the growth rate. To this end, we will quantify the so-called “interaction effect” between the growth rate (μ) and the type of mutation carried out by the strain (see Table 3).

**Table 3:** Number of genes differentially expressed according to the statistical criteria of interaction between the growth rate (μ) and the type of mutation carried out by the strain (wild-type and Δ*cobB* or Δ*patZ*).

**Contrast**	**Interaction 1 (μ and Δ*cobB*)**	**Interaction 2 (μ and Δ*patZ*)**
Genes with positive interaction	12 (3.0)	2 (5.0)
Genes with negative interaction	38 (3.1)	12 (5.0)
Total	50	14

*The variables are considered on two levels, high (+) or low (−), such that μ^+^ = batch (exponential-phase, non-limited culture), μ^−^ = chemostat (limited culture); strain^+^ = wild-type, strain^−^ = defective mutant. The values in parentheses indicate the associated false discovery rate (%FDR). For details see Tables S5 and S6*.

Note that according to the concept of super-ratio (Guebel et al., 2016), a positive interaction effect in Table 3 refers to those genes for which the change in the level of their transcription during the transition from the wild type to the indicated mutant in the glucose-limited chemostat (D = μ = 0.23 h^−1^) was significantly greater than the change in the transcription for the same type of transition but in batch culture (exponential phase, μ_max_ = 0.62 h^−1^). Thus, from Table 3 we conclude that there is a synergy between glucose limitation and some acetylated (or succinylated) transcription factors (or regulators), because the transcription of 12 genes appears as increased in the mutant Δ*cobB* (i.e., when deacetylase and desuccinylase enzyme CobB is absent).

Interestingly, from the 12 genes with positive interaction between μ and Δ*cobB*, multiple functions are covered in a quite coherent mode, because all contribute to supporting better survival under glucose limitation. Thus, three genes are associated with improving the influx of nucleotides (*codB, nupC, uraA*) and two genes are related to the nucleotide balance through the salvage pyrimidine pathway *(codA* and *ndk* genes, which codify the enzymes cytosine deaminase and nucleoside kinase, respectively). Another important set of genes is given by the *nuoM* gene (codifying a protein involved in the generation of proton-motive force, a member of a terminal respiratory chain), the *glnL* gene (codifying the regulatory protein *ntr*B, a member of the two-component system that senses the availability of nitrogen), the *puuB* gene (codifying an oxidase involved in the degradation of the polyamine putrescine), and the *mupC* gene (which codifies a siderophore involved in the uptake and reduction of ferric ions).

Conversely, a negative interaction denotes that the variation in the expression during the transition from the wild type to the indicated mutant in batch culture (exponential phase, μ_max_ = 0.62 h^−1^) is significantly greater than the one observed in the limited chemostat (D = μ = 0.23 h^−1^). Interestingly, in the case of negative interaction between μ and Δ*cobB*, 24 out of 38 genes detected (63%) codify non-annotated products (i.e., hypothetical proteins). From the remaining genes, 5 out of 38 (13%) correspond to non-codifying RNA (*micC, ryjA, sibd, ffs*, and *spf*), and 2 out of 38 (5.2%) genes detected correspond to opposing functions (*fliR* and *yjfO*, related respectively to the flagellar secretory protein system and to biofilm formation). More importantly, 4 out of 38 (10.5%) genes detected have very related functions, as they belong to a same operon, *prp*BCDE, which codifies enzymes corresponding to the propanoate cycle. Paradoxically, we have to conclude that the transcription of the set of genes with negative interaction appears as diminished under glucose limitation (or enhanced at the exponential phase), also due to the presence of some acetylated or succinylated transcription factor or regulator. In fact, these dual, opposing effects seem to be mediated by the same acetylation mechanism (this point is further analyzed in section Transcriptome Profiling of CRP Mutants).

Concerning the positive interaction between μ and Δ*pat*Z, only two genes were detected: one is *pyrD* (codifying the enzyme dihydroorotate dehydrogenase 2) and the other is *fhuF* (codifying a ferric reductase enzyme). By contrast, 12 genes were detected concerning the negative interaction between μ and Δ*pat*Z (Table 3). In this case, 9 out of 12 (75%) genes detected still have no annotated function (hypothetical proteins), while 2 out of 12 (17%) genes correspond to non-coding mRNA (*ryjA* and *ffs*). In addition, the *nrfG* gene was detected, which codifies dual enzyme (formyl-dependent nitrite reductase activity, in anaerobic metabolism) and heme lyase from the *nrf* EFG operon (for the insertion of heme into c552 cytochrome).

The data in Table 3 suggest a low transcriptional responsivity to the deletion of the enzyme PatZ in relation to the observed with the deletion of the enzyme CobB. While the mutant defective in the deacetylase CobB yielded a total of 50 differential genes, the mutant defective in the acetyl-transferase PatZ only accounted for 14 differential genes (see Table 3). The prevalence of CobB upon PatZ effects (ratio = 3.6:1) indicates that there is no symmetrical effect, even though the two enzymes exert opposing actions. This fact suggests two alternatives: (i) it could be possible that other, as yet unidentified acetyl-transferases are present; (ii) more feasibly, the prevailing mechanism of protein acetylation does not seem to be dependent on acetyl-transferase PatZ, but mediated non-enzymatically by acetylphosphate (Kuhn et al., 2014; Wolfe, 2016).

### Dissecting the transcriptional profiles in the ΔcobB and ΔpatZ strains

**Figure 3:**
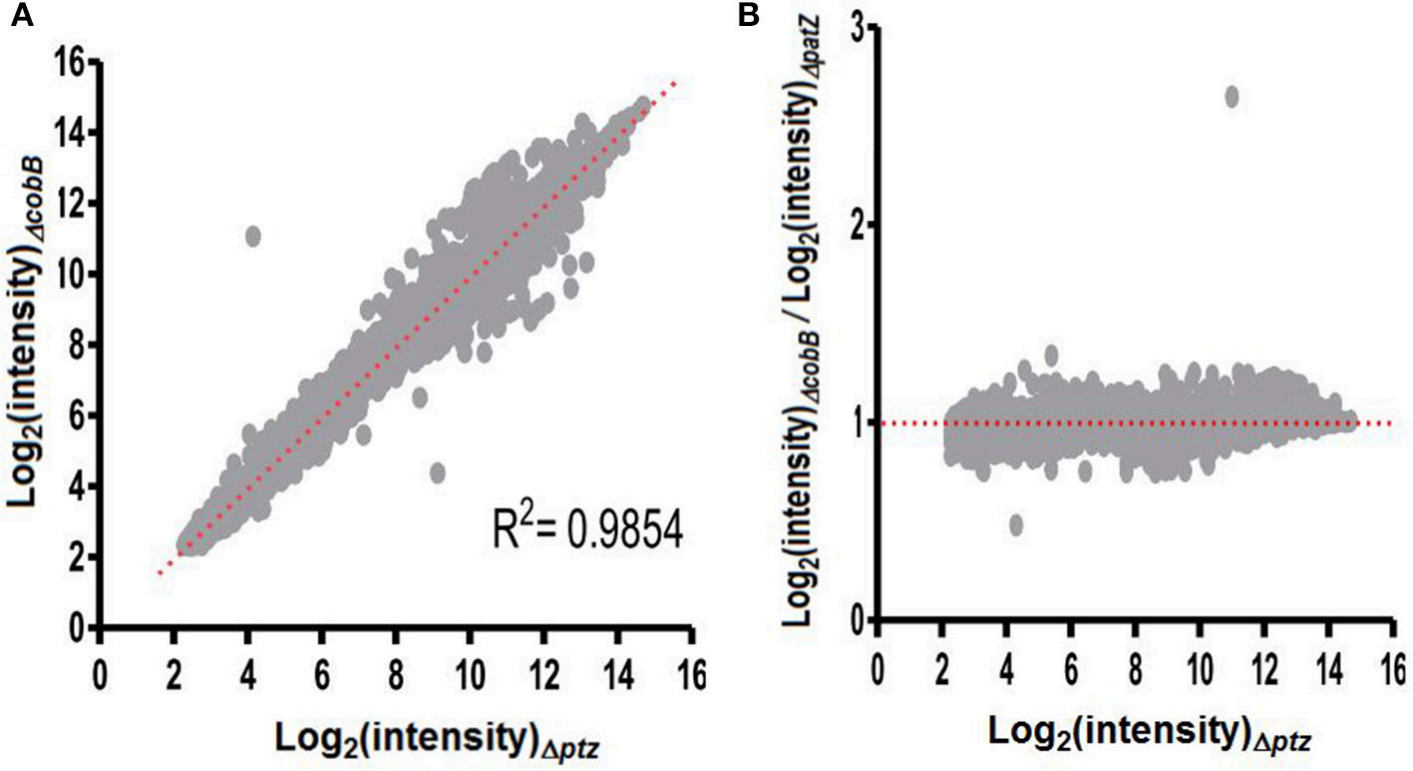
Relationship between the profiles of expression corresponding to both *E. coli* mutant strains when cultured in chemostat. **(A**) Linear relation between the expression levels of each gene belonging to the strain, whether it belongs to the Δ*patZ* (deficit in acetyl-transferase) or to the Δ*cobB* (deficit in deacetylase). **(B)** A ratio in function of the log_2_ (intensity)_Δ*patZ*_ where the ratio is given by quotient between the log_2_ of the transcript intensities of Δ*cobB* and Δ*patZ*.

Another result arising from our analysis is the verification that transcription profiles kept a very strong correlation between the two mutated strains tested along the more than eight thousand genes examined (*R*^2^ = 0.9854, see Figure 3A). Moreover, the regression over the cloud of points in Figure 3A has a linear trend with a unitary slope (*m* = 1.0113). This identity line is somewhat unexpected, because the mutant strains were designed to produce opposing effects on the acetylation-deacetylation phenomena, which is not evident from the monotonic, positive relation observed in Figure 3A. The expected relationship probably cannot be observed because it is masked as a consequence of the scant number of differential genes involved. So, the challenge is to identify a small number of altered genes by isolating them from a large series of genes affected by normal, noisy transcription.

The fact that the number of genes compromised by one or the other mutation is actually small in relation to the large proportion of genes that do not vary significantly suggests that data series corresponding to one of them, e.g., the mutant Δ*patZ*, can also be used as background to determine the differential genes in both mutants if, in addition to itself, the other mutant were also considered under the form of a ratio of expressions (see Figure 3B).

In the case of Figure 3A, the conceptual problem of determining which points (i.e., genes) depart significantly from the slope value = 1 is analytically solved by Q-GDEMAR. Using this method, we can establish the profile of distribution of the ratio between the expressions of Δ*cobB* and Δ*patZ* mutants, while making the deconvolution of central data distribution (see column 1, Table 4). In the case of Figure 3B, based on the similarity of this representation with Shewart and Deming's control charts (Tague, 2005), the dysregulated genes can be determined by establishing which points of the graph exceed the control limits (see column 2, Table 4). Importantly, whichever the type of discrimination procedure adopted, given that both data sets of the mutants came from chemostats operated at the same dilution rate, the differential genes are identified herein on the basis of equal influence of the growth rate, and without using information corresponding to the wild-type strain. This point is another important difference with respect to previous analyses done by Castaño-Cerezo et al. (2014).

**Table 4:** Number of differentially expressed genes according to different approaches to deal with the discrimination variables analyzed (Δ*cob*B:Δ*pat*Z ratio).

	**Distribution of Δ*cobB*:Δ*pat*Z ratio**	
	**Confidence limits with central data deconvolution**	**Confidence limits without central data deconvolution**
Genes up-regulated	94 (2.4)	22 (1.1)
Genes down-regulated	87 (2.7)	11 (2.2)

*The data came from the chemostat culture mode. The values in parentheses indicate the false discovery rate (%FDR). For details see Table S7 and Table S8*.

As expected, the analysis of the Δ*cobB*:Δ*pat*Z ratio by the deconvolution of central data of the distribution renders a much more sensitive detection of genes associated with the mutations tested. In fact, by applying Q-GDEMAR we were able to identify 94 up-regulated genes and 87 down-regulated genes, whereas the second procedure only detected 22 and 11 genes, respectively (see Table 4).

However, by comparing the up-regulated genes from column 1 in Table 4 with the up-regulated genes from column 1 in Table 2, we verify that all 94 genes from Table 4 (based only on the mutants) are actually included in the list of 104 genes previously detected in Table 2 (based only on the wild-type strain). Noticeably, this means that 100% of the genes identified as differentially up-regulated in column 1 of Table 4 cannot be explained by the mutation Δ*cobB*, as is supposed, but due to the low growth rate generated by the substrate limitation in the culture. Instead, from the 87 down-regulated genes detected in column 1 of Table 4, 96.5% (84 genes) differ from the ones detected in column 1 of Table 2 with the wild-type strain, and hence, they can be reliably attributed to an increased transcription under the mutation Δ*patZ*.

By applying the second strategy (confidence interval to the fluctuation of the Δ*cobB*:Δ*patZ* following the form of representation shown in Figure 3B), we recover those genes that depart significantly from the horizontal reference line (y = 1). In this way, as shown in column 2 of Table 4, we were able to identify 22 genes (FDR = 1.08%), whose transcription is apparently associated with the loss of the gene *cobB*, while 11 genes (FDR = 2.18%) showed a transcription that is apparently associated with the loss of the gene *patZ* (see Table S5, Supplementary Materials).

Importantly, the transcriptional effects associated with Δ*cobB* mutation that could not be previously identified from column 1 of Table 3 can now be identified from column 2 of Table 3. In fact, only 3 out of 22 genes (13.6%) detected in column 2 of Table 3 are shared with the list of up-regulated genes from column 1 of Table 1. That is, following the analysis over the distribution of the second ratio form in Table 3, it has been possible to identify 19 genes whose expression is enhanced by the loss of CobB activity. Interestingly, none of the 11 genes detected as down-regulated in column 2 of Table 3 are common to the list of 87 genes detected as down-regulated in column 1 of Table 3. Consequently, they have to be added to the list of 84 genes whose expression is enhanced by the loss of PatZ, thus bringing the total to 95 genes.

### Transcriptome profiling of CRP mutants

When we studied the CRP mutants, an additional problem arose, because the mutated genes cannot be cloned at their native position and the genomic insertion had to be carried out into the *paaH* locus. For this reason, the mutant ΔN was designed, which has to be considered as an additional control besides the wild-type strain (see Materials and Methods). In fact, the change of position in the cloning of native *crp* gene led to a considerable distortion, as 281 genes ended up being spuriously transcribed (see Table 5).

**Table 5:** Number of genes spuriously transcribed due to the insertion of the normal gene *crp* in the place of the gene *paaH*.

**Genes transcribed**	**Exponential phase**	**Stationary phase**	**Total false cases**
False negative (WT-ΔN >> 0)	22 (5.0)	47 (5.0)	69
False positive (WT-ΔN << 0)	191 (5.0)	21 (5.0)	212
Total false cases	213	68	281

*WT, wild-type strain; ΔN, positional crp mutant. The values in parentheses indicate the false discovery rate (%FDR)*.

Note in Table 5 that when the values of WT-ΔN depart significantly from zero, this means that the transcriptional response of the ΔN strain differs from the wild-type strain. Accordingly, when WT-ΔN << 0, this implies an increased transcription of some genes in the mutant ΔN with respect to what is observed in the wild-type strain. By contrast, when WT-ΔN >> 0, this implies a significant attenuation of transcription of some genes in the ΔN strain with respect to the wild-type strain. In spite of the significant number of over- and under-transcribed genes that appeared in Table 5, the validity of the experiments done with the *crp* mutants is still guaranteed. In fact, we have verified that the signal ratio (ΔN_stationary_/ΔN_exponential_) kept a high correlation with the homolog ratio (WT_stationary_/WT_exponential_) across the 8,862 genes tested (*R*^2^ = 0.8241, see Figure S1).

Thus, once we had identified the genes involved in Table 5 (see Table S9), appropriate corrections were applied to the list of differential genes corresponding to the CRP mutants. This was done by crossing the cases of “false negatives” from Table 5 against the list of total differentially down-regulated genes, and the cases of “false positives” in Table 5 against the list of total differentially up-regulated genes. In turn, the comparisons were disaggregated according to the phase of culture (exponential or stationary) and the form of Δ*crp* considered (Q-type or R-type). The net results after these corrections are shown in Table 6.

**Table 6:** Number of differential probes detected along the batch culture as caused by the Δ*crp* mutants in Lysine 100 (R-type: similar to constitutive deacetylated form, Q-type: similar to constitutive acetylated form).

**Mutant**	**Induction effect**	**Batch culture**	
		**Exponential phase**	**Stationary phase**
Δ*crp*(R-type)	Positive	472 (3.1)	503 (1.9)
	Negative	203 (3.7)	NDAS^*^
Δ*crp*(Q-type)	Positive	124 (3.0)	40 (4.3)
	Negative	60 (5.0)	78 (3.3)

*The values in parentheses indicate the associated false discovery rate (%FDR)*.

* *NDAS, not detectable as significant*.

Although we tested different types of discrimination variables to identify the transcriptional effect of CRP mutants, the best form that we found is the one applied in Table 6. There, the discriminating variable is given by the ratio between the signals from the analyzed mutant with respect to the ones in the wild type for each culture phase. This implies considering the following set of partial ratios: (R/WT)_stationary_ >>1 and (R/WT)_stationary_ <<1, (R/WT)_exponential_ >>1 and (R/WT)_exponential_ <<1, (Q/WT)_stationary_ >>1 and (Q/WT)_stationary_ <<1, (Q/WT)_exponential_ >>1 and (Q/WT)_exponential_ <<1.

The operation of correction applied to achieve the results shown in Table 6 proved particularly important for the data corresponding to the exponential phase. There, we had to exclude 46.9% of up-regulated genes for Δ*crp*(Q-type), 15.3% of up-regulated genes for Δ*crp(*R-type), and 13% of down-regulated genes for Δ*crp*(Q-type). However, only minor corrections were necessary for the remaining cases: 5.6% of down-regulated genes for the Δ*crp*(R-type) under exponential phase and 1.3% of down-regulated genes for Δ*crp*(Q-type) under stationary phase. No correction was needed for the up-regulated genes for Δ*crp*(Q-type) under stationary culture phase. The detail of the genes supporting Table 6 is given in Tables S10–S13.

**Figure 4:**
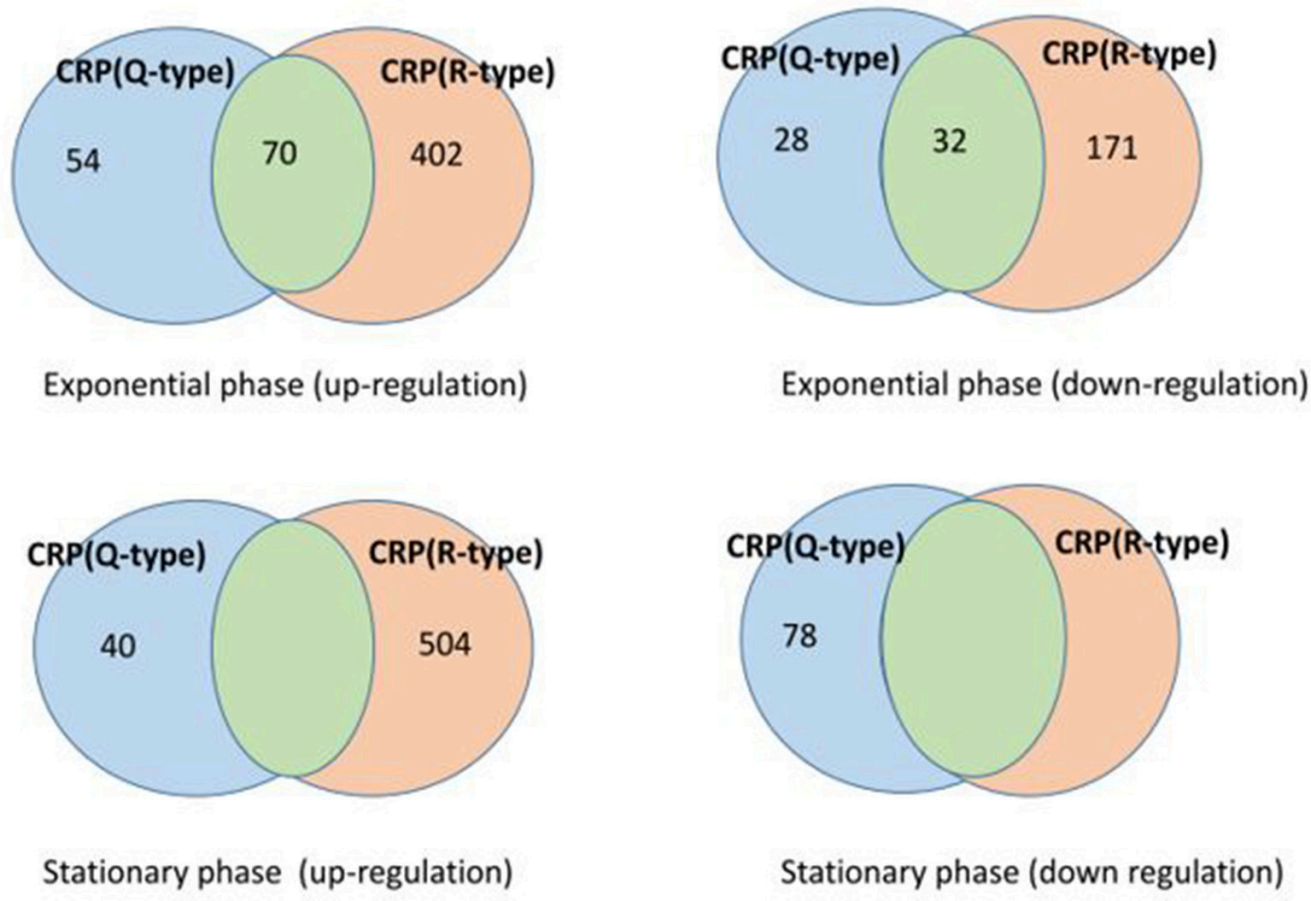
Venn diagram showing the disaggregation of the differential genes corresponding to each of the CRP mutants (Q-type: acetylated form, R-type: deacetylated form) according to the batch culture phase (exponential or stationary). The blue areas in the diagrams correspond to genes that depend exclusively on the Q-type, while the pink areas correspond to the genes that depend exclusively on the R-type. Importantly, the green regions correspond to common genes that can be influenced by both types of CRP mutants.

Importantly, based on data in Table 6 we were able to establish which genes are influenced by each type of mutant in each growth phase (see Figure 4).

From Figure 4 we observe several noticeable findings: (i) CRP is shown to act not only at the stationary phase when glucose is exhausted (bottom of Figure 4), but also during the exponential phase when glucose availability is unconstrained (top of Figure 4); (ii) For a given growth culture phase, both deacetylated and acetylated stages of CRP are shown to influence “exclusive” sets of genes (for the genes corresponding to blue and pink areas in Figure 4, see Tables S10–S13); (iii) However, in three out of four cases analyzed, each of the Δ*crp* mutants (Q-type and R-type) is shown to be able to produce “exclusive” effects in opposing directions (for a given culture phase in the Venn Diagram, compare areas from the left where genes are up-regulated with the homologs at the right where genes are down-regulated). Therefore, from the type of analysis done, we have to conclude that the acetylation stage of CRP by itself does not determine univocally the type of transcriptional effect produced; (iv) Importantly, at the exponential phase we detected two sub-sets where the genes seem be equally responsive to Δ*crp*(Q-type) and Δ*crp*(R-type) (see green areas of intersection, Figure 4). The identity of these common genes changes whether the genes are up- or down-regulated (see Tables S10, S11); (v) According to the number of genes affected, the transcriptional effects of CRP^deacteylated^ prevail over CRP^acetylated^ during the exponential phase (the ratio Δ*crp*(R-type):Δ*crp*(Q-type) ranged between 7:1 and 2.5:1, whether genes are up- or down-regulated, respectively). This feature is even more manifest at the up-regulated genes of the stationary phase, where the same ratio reached a value of 13:1; (vi) However, only the Δ*crp*(Q-type) was detected to exert inhibitory transcriptional effects in the stationary phase (see Figure 4).

### CRP effects independent of deacetylation/acetylation

The evidence that both mutants, Δ*crp*(Q-type) and Δ*crp*(R-type), shared some common target genes (13.3% in the up-regulated genes, 16% in the down-regulated genes, Figure 4) during the exponential phase was somewhat unexpected. A first, simple interpretation of this phenomenon could suggest that transcription of these sets of genes does not depend on the acetylation stage of CRP, but rather on the presence of several *conformer complexes* between c-AMP and CRP (Heyduk and Lee, 1989; Mukhopadhyay et al., 1999; Tworzydło et al., 2005; Tutar, 2008; Saha et al., 2015).

Supporting the “conformers” hypothesis, it is noticeable that neither the *crp* gene nor the *cyaA* gene was responsive to the acetylation stage of the CRP protein. In fact, the microarray probes corresponding to these genes displayed similar signal intensity under all the scenarios tested (data not shown). This point is important, since the CRP protein can influence its own transcription from the *crp* gene, while the *cyaA* gene is a direct CRP target that codifies de enzyme adenylate cyclase. This enzyme is required for the biosynthesis of cAMP, a metabolite that modifies allosterically the CRP protein such that CRP can behave effectively as a master regulator (Zheng et al., 2004; Zhou et al., 2014).

Thus, the above observations raise the question as to whether the regulation by acetylation, although conserved from bacteria to eukaryotes, actually has some form of hierarchical order. In this regard, the sub-sets of genes that are influenced by both CRP^deacetylated^ and CRP^acetylated^ (green areas, Figure 4) could represent distinct adaptations along the evolution and/or could have a distinct functional value than those genes preferentially affected by one or the other form (blue and pink areas, Figure 4). In any case, it is clear that *crp* and *cyaA* genes, due to their central role, are at the top of the CRP-dependent regulation cascade (see **Figure 6**). Other possible explanations for the shared genes between acetylated and deacetylated stages of CRP will be presented in the next section.

### Ontology analysis of CRP mutants

Tables S10–S13 provide exhaustive lists of the differential genes transcribed by the CRP mutants tested, whilst a global summary of this information is shown in Figure 3. By means of ontology analysis, we attempted to determine whether the differential genes identified can be associated with some specific bacterial function in terms of well-standardized, significantly over-represented categories (Rhee et al., 2008; Lin and He, 2012). For the ontology analysis we also adopted as cut-off an FDR value ≤5%. The results of these analyses are summarized in Tables S14–S18.

Some of the well-defined functional groups that we identified are associated with the stationary phase and are driven mainly by the Δ*crp*(Q-type) mutant. There, within the up-regulated genes, we detected a set of five enzymes related to the metabolism of Arginine (FDR = 1.8 × 10^−6^, see Table S16), whereas within the down-regulated genes, we identified a set of eleven ribosomal proteins (FDR = 1.2 × 10^−8^), a set of eleven enzymes related to the pyrimidine metabolism (FDR = 4.9 × 10^−5^), and a set containing seven H^+^-ATPases, which are related to the generation of energy in the oxidative phosphorylation (FDR = 1.1 × 10^−4^) (see Table S17). Interestingly, the same set of five enzymes related with catabolism of arginine detected for the stationary phase of batch culture based on the Δ*crp* mutants (Table S16) also appeared as significant in the ontology analysis of the stationary phase with the wild-type strain (experiment B in Table 2, ratio >>1).

Although other functional classes containing multiple related genes have also been recognized, including some associated with the Δ*crp*(R-type) mutant, these functional groups did not satisfy the FDR criterion (see Tables S14, S15). Given that in some of the conditions assayed, we identified 400–500 differential genes (see Figure 4), the low number of genes associated with the ontology classes and the low number of functional categories recognized as significant seems be a challenging problem.

However, this ontology picture can be explained by several factors: (a) CRP is a “master regulator.” As such, CRP interacts with 47 local co-regulators and controls the induction of 22 transcription factors (Martínez-Antonio and Collado-Vides, 2003; Martínez-Antonio et al., 2008). This complexity allows CRP to influence up to 283 operons (Ishihama et al., 2016). Hence, the low detection of significant ontological classes could be due to the very high number of pleiotropic effects associated with CRP. In fact, the numerous effects of CRP might appear as “distributed” across multiple functional categories, rather than concentrated. As a consequence, the probability of detecting a given effect will be strongly diminished; (b) Results from the ontology analysis are sensitive to the low number of genes sampled. Although the lists of differential genes sampled contained a large enough number, in some of the experimental groups analyzed around 50% of the genes fall under the category of “hypothetical genes.” Since these genes are classified as “unidentified” or “unknown,” they are not accounted for in the computation of the hypergeometric probabilities, thus shortening the list of available genes; (c) For the Δ*crp* mutants at the exponential phase, the correction applied to the data, based on the (positional) control strain ΔN, proved to be excessively protective (47% of differential genes of the Δ*crp*(Q-type) mutant were excluded, see section Transcriptome Profiling of CRP Mutants). In fact, the criterion of exclusion used was based on comparing the degree of over-expression (or under-expression) in the ΔN strain with respect to that observed in the wild-type (WT) strain rather than on considering qualitatively if induction (or repression) occurred.

**Figure 5:**
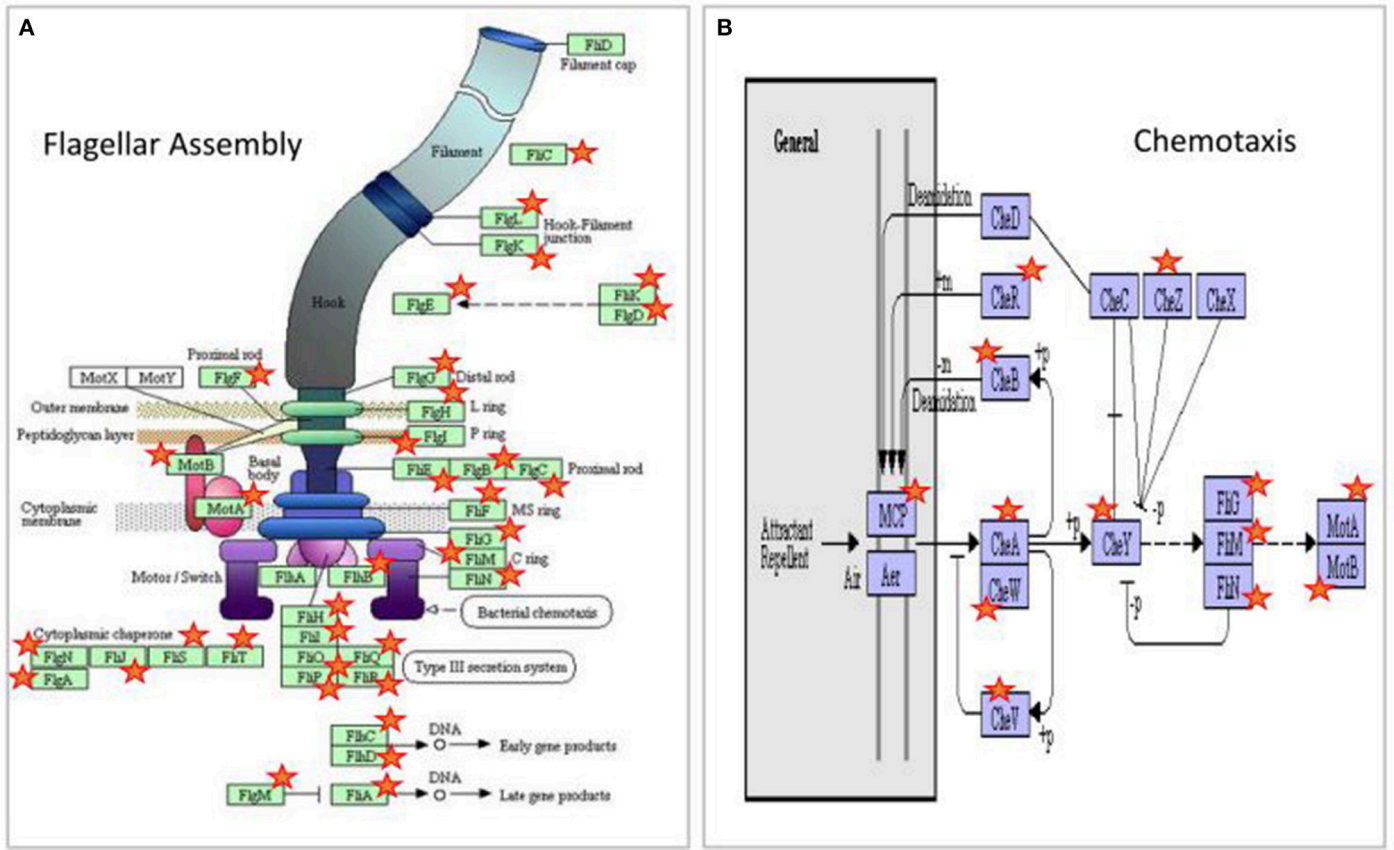
Up-regulated genes by CRP at the exponential culture phase associated with significant functional class over-representation in the ontology analysis. **(A)** Genes involved in flagellar assembly and motility (*n* = 32, FDR = 7.2 × 10^−44^); **(B)** Genes involved in chemotaxis and motility (*n* = 15, FDR = 1.8 × 10^−4^). MCP stands to a set of methyl-accepting chemotaxis proteins such as MCP-I (*tsr* gene), MCP-II (*tar* gene), MCP-III (*trg* gene), and MCP-IV (*tap* gene). The MCP proteins receive specific signals of chemical attractants and repellents, transducing them toward the Che proteins system and switch proteins (genes *fliG, fliM, fliN*), which finally modulate the motor activity of the flagella (genes *motA/B*). Whilst the master regulators FlhC and FlhD belong to the group of dysregulated genes induced by Δ*crp*(R-type), all the remaining dysregulated genes shown are up-regulated indistinctly by Δ*crp*(Q-type) as well as by Δ*crp*(R-type). All the dysregulated genes are marked with a red star. Diagrams are reproduced with permission from KEGG Pathways (maps02040 and 02030).

**Figure 6:**
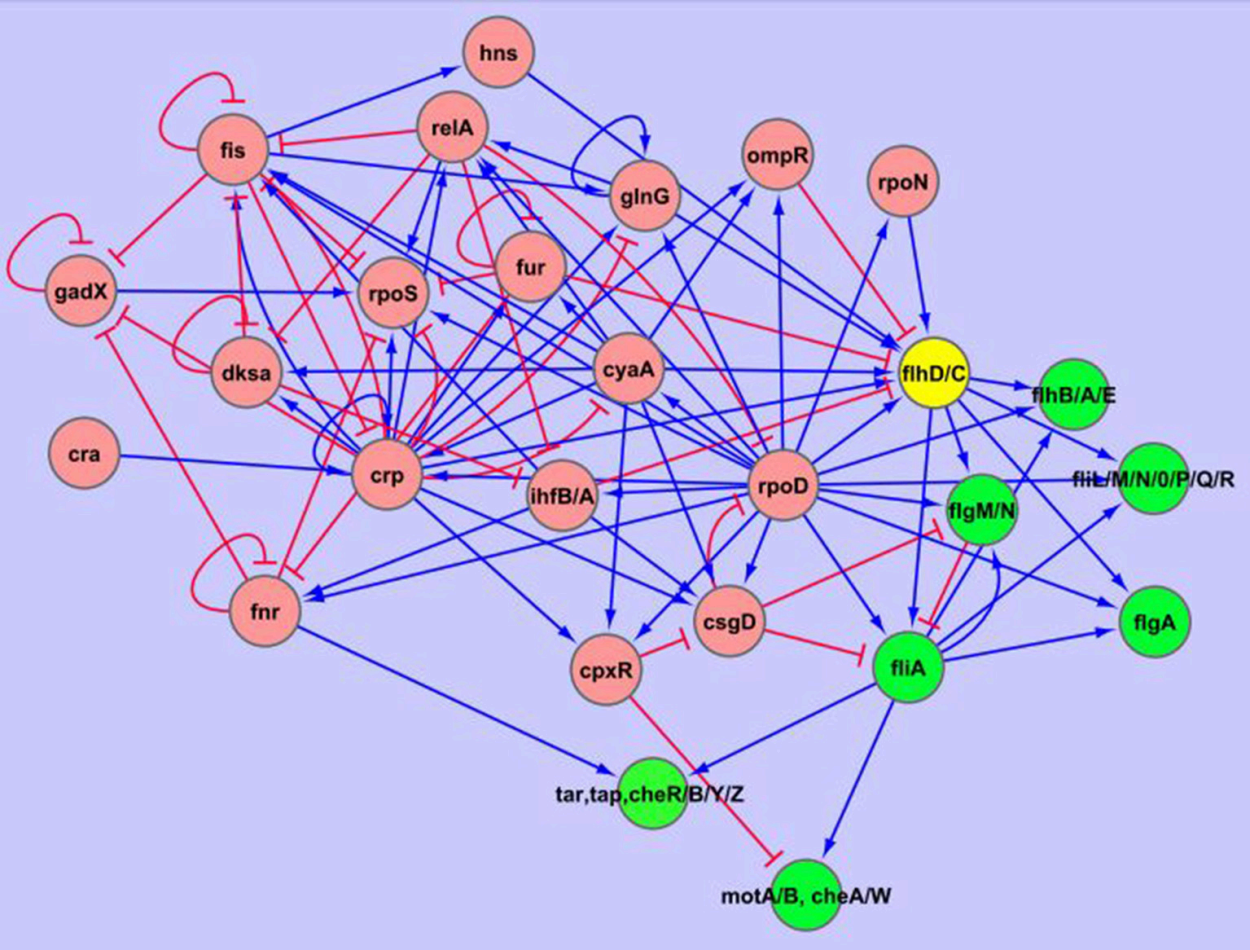
Genetic regulatory network (GRN) showing how the operons codifying the flagellar components and the chemotaxis proteins are under the control of CRP and several sigma factors σ^28^ (*fliA*), σ^54^ (*rpoN*), σ^70^ (*rpoD*). Genes and operons are represented as nodes. The directed edges represent the occurrence of a verified influence from the source gene on the target gene, such as positive (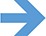) or negative (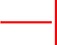) effects. The chemical intermediaries responsible of the effects among the genes (transcription factors, cAMP, ppGpp) are omitted. The nodes colored in green define a virtual output layer with the flagellar and chemotaxis genes. The yellow node, the operon *flh*D/C, codifies a master regulator with effects on several flagellar genes as well as on other important extra-flagellar genes (not shown; (Prüss et al., 2003)). The nodes colored in pink correspond to a highly regulated sub-network that include the genes codifying CRP, several sigma factors, and another interdependent regulators. The resulting signal from this complex layer acts as input toward the operon *flh*D/C and the remaining output genes. The diagram was built by composing the results showed in Figure 5 with the regulatory information given at EcoCyc database.

To overcome this problem, alternatively we only excluded the differential genes related to the *paa*ABCDEFGHIJK operon in which the *crp* gene was cloned. In this way, the number of up-regulated genes at the exponential phase, driven by Δ*crp*(Q-type) or Δ*crp*(R-type) indistinctly, increased from 70 to 104. More importantly, from this extended series we were able to verify the significant over-representation of 32 genes associated with the flagellar assembly (FDR = 7.2 × 10^−44^) together with another 15 genes associated with chemotaxis (FDR = 7.5 × 10^−15^) (see Table S18). Interestingly, most of the genes in this highly significant set are targets of the products codified by the genes *flh*C and *flh*D, which are responsive to the cAMP-CRP complex (see Figures 5, 6).

Reasoning in inverse form, the high significance detected herein for the flagellar and chemotaxis ontologies (FDR = 7.2 × 10^−44^) offers strong support for the existence of sets of genes with the apparent property of being driven indistinctly by Δ*crp*(Q-type) or Δ*crp*(R-type). In fact, the functional coherence of this group and the extremely low value of FDR ruled out the possibility of a methodological artifice or a grouping generated by chance.

However, several questions still emerge: (i) Given that a sub-set of genes appeared equally transcribed by the two opposing Δ*crp* mutants, one could wonder about the relevance of the acetylation stage of CRP for the transcription of these genes; (ii) In the contrary, assuming differences in the transcriptional effects associated to each Δ*crp* mutant: Might it be possible that the CRP acetylation variants acting at different levels of the CRP regulatory network lead to a situation in which the global effect appear “as if” no differences exist? (green areas in Figure 4); (iii) How are explained the “exclusive” differential effects also observed for each of the acetylated CRP stages? (blue and pink areas in Figure 4); (iv) Finally, it should be explained also how a given acetylated (or deacetylated) form of CRP can exert both positive and negative effects within a same growth phase (for a given row, areas with the same color between the left and the right diagrams in Figure 4). To address these questions we have compiled the information concerning the genes involved in Figure 6.

In this regard, it is known that the *crp* gene is regulated positively by the transcription factor CRA (FruR), but only if the level of fructose 1,6-biphosphate is low. The *crp* gene has a complex relationship with its negative regulator FIS, since CRP induces the *fis* gene when FIS is absent, but represses this gene when both are present together (Nasser et al., 2001). In addition, the *crp* gene has two binding sites for the CRP protein, a stimulatory one located upstream of the promoter, and an inhibitory one located downstream of the promoter (Ishizuka et al., 1994). CRP is not really what is bound, but rather the cAMP-CRP complex (Saha et al., 2015) (see Figure 6).

In turn, the availability of cAMP is a complex function of the *cyaA* gene activity and the energy status of the cell (levels of ATP, GTP, AMP). However, the *cyaA* gene is negatively regulated by the cAMP-CRP complex, while ATP can also be diverted toward the synthesis of the alarmone ppGpp mediated by the pyrophosphokinase RelA, but whose activity depends on the nutritional and stress stage of the bacteria (Magnusson et al., 2005; Burgos et al., 2017) (see Figure 6).

On the other hand, the assembly of flagella and the synthesis of components of the chemotaxis system during the exponential growth phase require the simultaneous transcription of a plethora of genes, distributed along non-contiguous operons. However, as is shown in Figure 6, most of these genes are not direct targets of CRP, but are induced by the molecules codified by the genes *flhD* and *fllhC*. Importantly, both genes belong to the same operon and are direct targets of the cAMP-CRP complex (see Figure 6).

Note that the transcriptional processes related to the flagellar biogenesis and chemotaxis are mainly mediated by RNA polymerases containing sigma factors σ^28^, σ^54^, and σ^70^. However, the transcription of these sigma factors is regulated by several repressors (ppGpp-DKSA complex, CSGD protein) and the protein CPXR (a repressor of the *csgD* gene). All these repressors have the notable particularity that they are induced by the cAMP-CRP complex (see Figure 6). Moreover, the IHFA-IHFB complex, which represses the critical genes *flhC* and *flhD*, has a dual effect on the genes *fliA* and *rpoF*, which codify the sigma factors σ^28^ and σ^70^, respectively. In fact, the IHFA-IHFB complex acts as an inducer of CSGD –a sigma factor repressor–, while simultaneously contributing to the induction of these sigma factors. In the same line, the IHFA-IHFB complex also leads to the induction of the factor anti-sigma 28 from the gene *flgM* (see Figure 6).

In brief, given the complex structure of the regulatory network involved in flagellar assembly, it is highly plausible that if a given acetylated form of CRP were able to influence a gene codifying a repressor negatively, while the other acetylated stage were able to influence the target gene of this repressor positively, then the gene in question could appear up-regulated as being modulated indistinctly by both opposing CRP species. Similarly, a gene could appear as down-regulated indistinctly by both opposing CRP species if one acetylated CRP stage acts positively on a gene codifying a repressor, while the other acetylated CRP stage acts negatively on the target gene of this repressor.

To illustrate this hypothesis, based on the network showed in Figure 6, the following chains of process leading to the synthesis of the FLHD-FLHC complex can be identified:

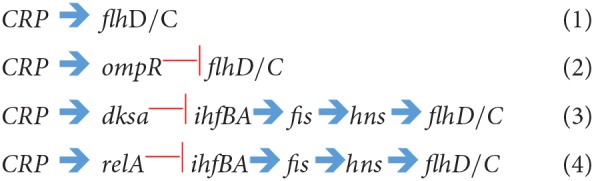



From these set of processes it turns evident that if it were required that the operon *flh*D/C be maximally transcribed, CRP in Equation (1) might be in a one of the CRP acetylation stages (the able to activate the operon *flh*D/C), whereas in Equations (2)–(4) CRP could be present in the contrary stage of acetylation (thus inhibiting the genes *ompr, dksa, relA*). This distribution of CRP variants would provide the maximal induction of the *flh*D/C operon, thus showing how is feasible that a gene appears from a phenomenological point of view as if they were equally influenced by CRP^acetylated^ and CRP^deacetylated^ even when this was not the real mechanism.

This hypothesis to be satisfied requires that both acetylated stages, CRP^acetylated^ and CRP^deacetylated^, coexist in some proportion. Moreover, both CRP^acetylated^ and CRP^deacetylated^ could have similar affinities for their DNA-binding sites but would differ in their mode to interact with the RNA polymerase. The first requirement does not seem restrictive since it depends only on the balance between the enzyme CobB (a deacetylase) with respect to the concentration of acetyl-phosphate (the main no-enzymatic donor of acetyl moieties, related with the metabolism of acetate). Consequently, the *flh*D/C operon could also be transcribed in variable extents under distinct biological scenarios according to the increased or decreased balance between these factors.

The fact that CRP^acetylated^ and CRP^deacetylated^ also showed to exert specific effects (see Figure 4) can be explained by assuming that each of CRP variant acts on a given target gene but not on the genes which codify a repressors of these target genes. The case that a given form of CRP can exert both, positive and negative effects on different genes is difficult to justify; it could require a better understanding of the interactions between CRP and other transcriptional regulators with action on these genes.

## Concluding remarks

The present study aimed to characterize the regulation by the acetylated/deacetylated forms of CRP in *Escherichia coli*. In this context, we also analyzed their dependence on their most direct regulator (glucose) and some direct effectors (deacetylase CobB, peptidyl N(ε)-Lysine acetyl-transferase PatZ). Although this topic belongs to the ambit of molecular physiology, the clarification of these mechanisms could have important consequences in the field of biotechnological applications (Kuczynska-Wiśnik et al., 2016; Ahsan et al., 2017; Basak et al., 2017; Ishigaki et al., 2017; Venkat et al., 2018), and even for some medical issues (Bernal et al., 2016; Ou et al., 2017).

Our first reflection is that effects of CRP are currently associated with the stationary phase of bacterial cultures. There, the catabolic repression exerted by CRP is relieved, thus enabling the activation of genes for alternative substrate assimilation and a better adaptation to this stage (Deutscher, 2008; Schilling et al., 2015). Apart from the catabolic repression phenomenon, we found few studies examining other effects of CRP in growing bacteria (Gomez-Gomez et al., 1996; González-Flecha and Demple, 1997; Shimada et al., 2013; Baron and Eisenbach, 2017; Zhou et al., 2017). A common feature of these studies is that they focused on a single gene or protein. Our analysis, based on the systems biology approach, lead us to analyse the CRP effects on the genome-wide scale with the intention of not only capturing the trends emerging from the data but also, when feasible, inferring the more general organizing principles behind the CRP regulation.

At this genome-wide level, we were able to verify that CRP acts along all the culture phases (i.e., with excess, limitation, or shortage of glucose). However, what was unexpected was our observation that successive transitions toward the stages of increasing glucose limitations appear to be associated with the up-regulation of specific sets of target genes rather than with the loss of genes present when glucose was in excess (Table 2).

One could conjecture at least two possible explanations: (i) most of the genes are essential to survival, with only a minor fraction responsible for the adaptation; (ii) the genes acquired during the glucose limitation could be an adaptive response to the stress generated by the carbon-source shortage, which would coexist with the pre-existing machinery if stress intensity is not extremely high. However, the first hypothesis must be ruled out, since <10% of the *Escherichia coli* genome is essential for its survival (Mahadevan and Lovley, 2008). Concerning the second hypothesis, it is well-established that the transcriptional response to “nutrient limitation” differs from the response to “deprivation” (Saldanha et al., 2004). Moreover, in the glucose-limited chemostat, we did not observe any dysregulation in the stress markers (e.g., *relA, rpoS, rpoE, rpoN, crp, arcA, arcB, katE, sodA, cpxa, cpxr*), nor did the transcription of genes codifying some enzymes of central metabolic pathways appear to be dysregulated (e.g., *pfka, aceA, aceE, aceF, aceK, sdhA, sdhB, sdhC, sucD, fumB, pykA, pykF, glpc, pta*).

Similar observations have been made for other biological systems. Thus, important variations have been verified in the fluxes that these enzymes catalyze, leading researchers to conclude that regulation under glucose-limiting conditions exists, but must be mainly of a post-transcriptional nature (Daran-Lapujade et al., 2004). In our case, these reports lend support to the possible importance of acetylation-deacetylation as the mechanisms responsible for the observed regulation. In fact, although the effects of glucose limitation overlap with the effects caused by the mutants Δ*cobB* and Δ*patZ*, by introducing Experiment A (Table 2), analyzing one mutant against the other, and applying the factorial analysis, we were ultimately able to identify the genes that really participate in the interaction among these variables, thus offering direct proof that regulation by acetylation/deacetylation is indeed operating (sections Substrate Availability and Genome-Wide Transcription, Interaction Between Growth Rate and Transcription Profile, Dissecting the Transcriptional Profiles in the ΔcobB and ΔpatZ Strains). Moreover, from the asymmetry observed in Table 4 between the total number of genes affected by the mutant Δ*cobB* and the ones in the case of the mutant Δ*patZ* (ratio 2.6:1), it was inferred that some as yet unidentified acetyl-transferase must exist, or that a non-enzymatic mechanism (e.g., acetyl-phosphate) might be the dominant mechanism with respect to the mediated by the enzyme PatZ. This investigation highlight some aspects that requires further inquiry. This is the case of the propanoate pathway in the wild-type strain under glucose limitation, the observed dysregulation of non-coding RNAs genes under negative interaction between the growth rate and the Δ*cobB* mutant (genes *micC, sibD, ryjA*, and *spf*), and the dysregulation of genes codifying small RNAs under the negative interaction between growth rate and the Δ*patZ* mutant (genes *ryjA* and *ffs*) (see section Interaction Between Growth Rate and Transcription Profile). Note that the non-coding RNA MicC, by modulating OmpC, can inhibit the bacterial motility, while the small RNA Spf inhibits the formation of the complex succinate:quinone oxide reductase from the operon *sdh*CDAB. The Ffs small RNA, together with the Ffh peptide, constitute the signal recognition particle (SRP), which directs the insertion of the nascent proteins into the plasma membrane.

As a second comment, we conclude that the acetylation of CRP has proved to be a no ordinary form of regulation. In fact, both CRP^acetylated^ and CRP^deacetylated^ forms can produce positive and negative effects on “exclusive” sets of genes. The number and identity of these genes is conditioned by the glucose availability (section Transcriptome Profiling of CRP Mutants). Specific ontology functional classes appeared to be associated with each of these different sets of genes (section Ontology Analysis of CRP Mutants).

Apart from the exclusive differential effects recognized for each deacetylated/acetylated stage of CRP in a given culture phase, we also identified groups of genes that, being directly or indirectly responsive to CRP, seem not to depend on the CRP acetylation stage. For these genes, susceptible to “shared” effects, we proposed two possible explanations. One is that their regulation does not depend on the acetylation stage of CRP but rather on the conformational stage of the cAMP-CRP complex (section CRP Effects Independent of Deacetylation/Acetylation). Alternatively, these groups of genes might depend on each acetylation stage of CRP, but due to the nested characteristics of the network involved, phenomenologically the regulation occurs as if the observable mechanisms were independent of the acetylation forms (section Ontology Analysis of CRP Mutants). Although in sections Transcriptome Profiling of CRP Mutants, CRP Effects Independent of Deacetylation/Acetylation, Ontology Analysis of CRP Mutants we presented the arguments supporting each of these hypotheses, additional experiments will be required to rule out one or the other. In any case, from the ontology analysis we determined that the assembly of flagella and chemotaxis are the most important functions that, being regulated by the cAMP-CRP, appeared as being equally sensitive to both CRP^acetylated^ and CRP^deacetylated^ (FDR = 7.2 × 10^−44^) (section Ontology Analysis of CRP Mutants). Our finding of an enhanced transcription of the genes corresponding to the flagellar assembly and chemotaxis during the exponential phase is in agreement with some published reports (Amsler et al., 1993; Sim et al., 2017), in contrast with the generally accepted conception that flagellar activity is associated to the stationary phase.

Recently, it has been suggested that the Δ*crp*(Q-type) mutant (i.e., the CRP^acetylated^) exerts an inhibitory effect on the CRP-driven genes containing the type II promoters, while possibly also simultaneously exerting a positive effect on the CRP-driven genes containing the type I promoters (Davis et al., 2018). However, the hypothesis of the opposed effect of CRP^acetylated^ depending on the type of promoter still remains to be experimentally verified. The most relevant conclusions of this study arose from an *E. coli* in which the mutated genes Δ*crp* were cloned in the chromosomal gene *paaH* and challenged with glucose and acetate. In our investigation we have data concerning to this genetically modified strain with glucose as source of carbon (see section Strains and Conditions Tested). We have considered the data referred to the Δ*crp* mutants with glucose because we verified that the strain ΔN (the cloning control) exhibits an acceptable transcriptional behavior when compared with the wild-type strain (*R*^2^ = 0.8241, see section Transcriptome Profiling of CRP Mutants). However, the same strain ΔN showed to be no-appropriated when is challenged with acetate. In this case, we observed that the transcriptional response of the strain ΔN differs markedly from the one observed in the wild type strain when tested along 8,862 genes (*R*^2^ = 0.4406, see Figure S1). In any case, beyond of the critical experimental details mentioned, the theory presented by Davis et al. (2018) does not seem to assign any role to the Δ*crp*(R-type) mutant (i.e., the CRP^deacetylated^) for which we have also observed specific, significant effects. Neither does the proposal account for the group of genes that we have herein detected as being equally influenced by both CRP^acetylated^ and CRP^deacetylated^.

Other researchers, using advanced computational techniques, have built a predictive model of CRP based on the DNA sequences of its binding sites in the *E. coli* genome (Tsai et al., 2018). This model not only accounts for the induction effects of CRP-responsive genes with promoters of the type I and type II, but also predicts the repression effects of CRP. It could seems then that acetylation/deacetylation of CRP has not any regulatory influence. However, despite the importance of this model, since it is based uniquely on descriptors derived from the sequence of DNA, it cannot deal with questions such as the physiological conditions under which CRP will exert a positive or a negative effect. To be able to address this question it would be required to know the factors beyond of the DNA sequence that determine the binding of CRP to a positive or negative site in a given gene. In turn, this requires to establish if the binding of CRP to a positive or negative sequence in the specific DNA sites studied is a stochastic phenomenon or it depends on some particular form of post-translational modifications in the CRP or in another associated transcriptional regulators (e.g., acetylated/deacetylated; Brown et al., 2017). This question is relevant because we have observed that there is not a simple binary correspondence between acetylated/deacetylated form of CRP and their positive or negative effects. Both forms of CRP can produce both positive as negative effects during the exponential phase as well as at the stationary phase (see Figure 4).

Finally, with a view to future experiments, we have to mention the need to consider the succinylation phenomenon, since the deacetylase enzyme CobB also exerts a desuccinylase action (Colak et al., 2013), while succinylation as a post-translational modification overlaps with many sites of peptidyl N(ε)-Lysine acetylation (Weinert et al., 2013b; Okanishi et al., 2017). Hence, this information could help to clarify or even to modify our comprehension of regulation by acetylation/deacetylation in *E. coli*.

## Author contributions

DG designed and performed the analysis of the data. NT supervised the data analysis. DG and NT wrote the manuscript.

### Conflict of interest statement

The authors declare that the research was conducted in the absence of any commercial or financial relationships that could be construed as a potential conflict of interest.

## Footnotes

^1^https://www.ncbi.nlm.nih.gov/geo/query/acc.cgi?acc=GSE96955

## Supplementary material

Figure S1 Comparison between the overall transcriptional responses of the ΔN strain (cloning control) with respect to the one in the wild-type strain whether the type of substrate used (glucose or acetate).

Table S1 Differential genes transcribed in the wild-type strain of *E. coli* under glucose-limited culture (chemostat, D = 0.3 h^−1^) with respect to the one growing in glucose excess (exponential phase, μ_max_ = 0.62 h^−1^ in batch).

Table S2 Differential genes transcribed in the wild-type strain of *E. coli* in stationary phase (batch culture) with respect to the one growing in glucose excess (exponential phase, μ_max_ = 0.62 h^−1^ in batch).

Table S3 Differential genes transcribed in *E. coli* carrying defective mutation Δ*cobB* with respect to the wild-type strain, both growing in chemostat under glucose-limited conditions (D = 0.3 h^−1^).

Table S4 Differential genes transcribed in *E. coli* carrying defective mutation Δ*patZ* with respect to the wild-type strain, both growing in chemostat under glucose-limited conditions (D = 0.3 h^−1^).

Table S5 Differential genes transcribed in *E. coli* according to the interaction in the factorial 2 × 2, where the factors analyzed are the specific growth rate (μ high due to glucose excess in exponential phase or μ low due to the glucose-limited conditions in chemostat) and the mutation Δ*cobB* (absence or presence).

Table S6 Differential genes transcribed in *E. coli* according to the interaction in the factorial 2 × 2, where the factors analyzed are the specific growth rate (μ high due to glucose excess in exponential phase or μ low due to the glucose-limited conditions in chemostat) and the mutation Δ*patZ* (absence or presence).

Table S7 Differential genes using as discrimination variable the ratio between the expression in the two mutants (Δ*cobB*: Δ*patZ*) when applying the confidence limits arising from the Gaussian central region deconvolution (Q-GDEMAR).

Table S8 Differential genes using as discrimination variable the ratio between the expression in the two mutants (Δ*cobB*: Δ*patZ)* when applying the confidence limits on the whole distribution without Gaussian central region deconvolution.

Table S9 Differential genes spuriously transcribed when comparing the cloning-control strain (ΔN) against the wild-type strain (WT), by applying Q-GDEMAR to the difference (WT-ΔN) as discrimination criterion for the exponential phase and stationary phase of batch culture.

Table S10 Differential genes transcribed in *E. coli* carrying Δ*crp*(Q-type) mutation during exponential growth phase with and without correction by ΔN.

Table S11 Differential genes transcribed in *E. coli* carrying Δ*crp*(R-type) mutation during exponential growth phase with and without correction by ΔN.

Table S12 Differential genes transcribed in *E. coli* carrying Δ*crp*(Q-type) mutation during stationary growth phase with and without correction by ΔN.

Table S13 Differential genes transcribed in *E. coli* carrying Δ*crp(*R-type) mutation during stationary growth phase with and without correction by ΔN.

Table S14 Ontology analysis for the Δ*crp*(Q-type) and Δ*crp*(R-type) mutants in the case of up-regulated genes during the exponential growth phase.

Table S15 Ontology analysis for the Δ*crp*(Q-type) and Δ*crp*(R-type) mutants in the case of down-regulated genes during the exponential growth phase.

Table S16 Ontology analysis for the Δ*crp*(Q-type) and Δ*crp*(R-type) mutants in the case of up-regulated genes during the stationary phase.

Table S17 Ontology analysis for the Δ*crp*(Q-type) mutant in the case of down-regulated genes during the stationary phase.

Table S18 Detail of the genes up-regulated during the exponential growth phase that were induced by both the Δ*crp*(Q-type) and the Δ*crp*(R-type) mutants, being enriched in the flagellar assembly and chemotaxis ontology functions.
