# Supplementary material for: Influence of Glucose Availability and CRP Acetylation on the Genome-Wide Transcriptional Response of Escherichia coli: Assessment by an Optimized Factorial Microarray Analysis
Source: Front Microbiol. 2018 May 23;9:941. doi: 10.3389/fmicb.2018.00941 (PMC5974110; doi:10.3389/fmicb.2018.00941)
Supplement: Figure S1 — Comparison between the overall transcriptional responses of the ΔN strain (cloning control) with respect to the one in the wild-type strain whether the type of substrate used (glucose or acetate). [file Image_1.pdf]

Supplementary Materials

Influence of glucose availability and CRP acetylation on the genome-wide transcriptional response of *Escherichia coli*: assessment by an optimized factorial microarray analysis

Daniel V. Guebel[1] and Néstor V. Torres[2*]

[1]Biotechnology Counselling Services, Buenos Aires, Argentina; [2]Systems Biology and Mathematical Modelling Group, Department of Biochemistry, Microbiology, Cellular Biology and Genetics, Institute of Biomedical Technologies, Center for Biomedical Research of the Canary Islands, University of La Laguna, San Cristóbal de la Laguna, Spain.

*e-mail (NVT): ntorres@ull.edu.es

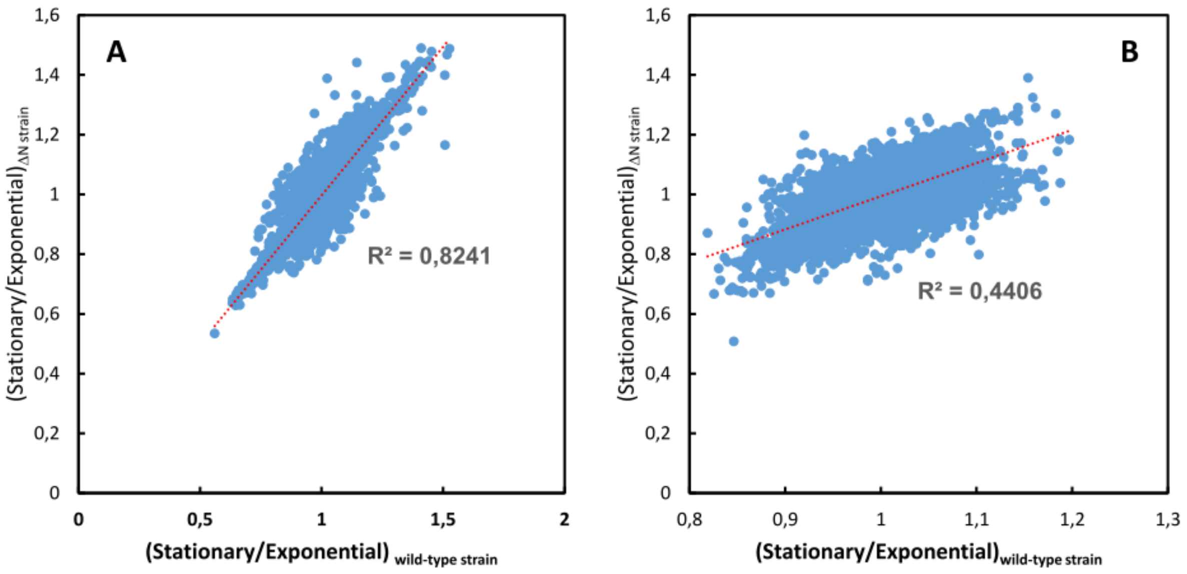

Figure S1: Comparison between the overall transcriptional responses of the ΔN strain (cloning control) with respect to the one in the wild-type strain whether the type of substrate used in the culture medium. **A.** Glucose used as source of carbon; **B**. Acetate used as source of carbon. The degree of similarity between the transcriptional responses in both strains is quantified through the value of the squared Pearson Correlation coefficient (R^2) after the ratio of signals corresponding to the stationary growth phase to the exponential growth phase for the ΔN strain is analysed as a function of the ratio of signals stationary growth phase to exponential growth phase for the wild-type strain along the total microarray data (n=8662 genes). The microarray data are taken from Gene Expression Omnibus (GEO dataset, accession code: GSE96955).
