# Supplementary material for: Influence of Glucose Availability and CRP Acetylation on the Genome-Wide Transcriptional Response of Escherichia coli: Assessment by an Optimized Factorial Microarray Analysis
Source: Front Microbiol. 2018 May 23;9:941. doi: 10.3389/fmicb.2018.00941 (PMC5974110; doi:10.3389/fmicb.2018.00941)
Supplement: Table S14 — Ontology analysis for the Δcrp(Q-type) and Δcrp(R-type) mutants in the case of up-regulated genes during the exponential growth phase. [file Supplementary_TABLE_S14.docx]

**Table S.14 | Main functional classes in up-regulated genes during exponential-growth phase according to the acetylation stage of CRP.**

| **CRP-dependence** | | | | | | | |
| --- | --- | --- | --- | --- | --- | --- | --- |
| **Functional**  **Classes** | **Exclusive on Q-Type** |  | **Functional**  **Classes** | **Exclusive on R-Type** |  | **Functional**  **Classes** | **Common to Q- and R-type** |
| Transcription  regulators | TtdR, *rhaS*, *yabN,*  Repressor ECs1862 |  | Transcription regulators & Cold shock proteins and | *flhD*, *rfAh*, *focX*, *hydG*, *yjcR* and emR (multi-drug resistence), *ncrdlD* and Isrc (non-coding RNA), N-Ac-glucosamine operon repressor, *cspA*, *cspB*, Ecs2601, Ecs4606, Ecs0399 |  | Transcription regulators | *flhC*, sgR, cl repressor protein, ECs 1862, |
| Transporters | Multi-drug efflux YjcQ, PTS-fructose transport *(fruB & frwC);* maltose transport  *(lamB*, *malE,*  & *malK ),*  permease for 3 nucleosides, *alaE* (alanine exporter), ECs3053, ECs3272, *yhpD* (permeases) |  | Transporters | *potF* (putrecsine), *gabP* (GABA), c4015 (ribose),  fepA, *fepE, chuA, cysG, Fiu*  *(*Ferric ion and heme transport),  Usher protein, phnC (phosphonate ester transporter) |  | Transporters | Muti-drug efflux Mdtu,  sorbose-permease PTS system IIB,  pheP and ECs0614 (phenyl-alanine transporters), permease ECs0621,  *entD (*Enterobactin), *fadL*(long-chain fatty acid transport) |
| Others | *fadE* (acyl-CoA dehydrogenase), *bioD* (dithiobiotin synthetase), MokW, peptidase yhbU,  *ylcG*, *rhaD*, *hlmC* (hemolysin C), *ureA* (urease subunit gamma), *dmSA* (anaerobic dimethyl sulfoxide reductase), N-metabolism (*nirB*, *napF*, *napD*) |  | Membrane Component | EivA, Escs, HopD, YdeE, Evga regulator,  Multidrug-efflux transporter, type III secretion system protein, transporters ECs0740 and ECs2610 |  | Flagellum Assembly (**FDR = 7.2 x10^-44^)** and Chemotaxis **(FDR = 7.5 x10^-15^)** | *flhC*, flhD, and their cascade of **30** down-stream cascade genes together with **11** chemotaxis genes (see Figure 4, and/or Tables 18 for the complete list) |
|  |  |  | Virulence | Ecs 1991, Ecs1236, Ecs1649 |  | Others | ECs5001 *(*PTS-sorbosel IIc), *mtdl*  (mannitol-1-P, 5-dehydrogenase), ECs1170 and ECs1020 (C4 Zn-type finger proteins), CesT (chaperone protein),  wzxE and ECs4725 (cytochromes) ,  NAD(P)H-dependent FMN reductase (ECs 1020),  isopentenyl-diphosphate delta-isomerase (ECs3761) |
|  |  |  | Periplasm | neutral amino-acid efflux protein; nickel/cobalt efflux protein RcnA, tyrosine kinase ECs2865, regulator ECs3249 |  |  |  |
|  |  |  | Fimbria/Cell Adhesion | FimF, FimG, Fimbrial-ike protein, Ecs4020,  c4214 |  |  |  |
|  |  |  | Others | *murD* (muramic acid synthesis), *agp*  (glucose-1P/inositolP  phosphatase), *gcvP* (glycerine dehydrog.), *mchF* (microcine secretion), *fcl* (GDP-fucose synthesis) |  |  |  |
