# Supplementary material for: Influence of Glucose Availability and CRP Acetylation on the Genome-Wide Transcriptional Response of Escherichia coli: Assessment by an Optimized Factorial Microarray Analysis
Source: Front Microbiol. 2018 May 23;9:941. doi: 10.3389/fmicb.2018.00941 (PMC5974110; doi:10.3389/fmicb.2018.00941)
Supplement: Table S15 — Ontology analysis for the Δcrp(Q-type) and Δcrp(R-type) mutants in the case of down-regulated genes during the exponential growth phase. [file Supplementary_TABLE_S15.docx]

| **Influence of glucose availability and CRP acetylation on the genome-wide transcriptional response of *Escherichia coli*: assessment by an optimized factorial microarray analysis**  Daniel V. Guebel^1^ and Néstor V. Torres^2*^  ^1^Biotechnology Counselling Services. Buenos Aires. Argentina; ^2^Systems Biology and Mathematical Modelling Group. Department of Biochemistry, Microbiology, Cellular Biology and Genetics. Institute of Biomedical Technologies. Center for Biomedical Research of the Canary Islands. University of La Laguna. San Cristóbal de la Laguna. Spain.  *e-mail (NVT): [ntorres@ull.edu.es](mailto:ntorres@ull.edu.es) |
| --- |

**Table S15 | Main functional classes present in the down-regulated genes during the exponential-growth phase according to the acetylation stage of CRP**

| CRP-dependence (down-regulated genes) | | | | | | | |
| --- | --- | --- | --- | --- | --- | --- | --- |
| Functional  Classes | Exclusive on Q-Type |  | Functional  Classes | Exclusive on R-Type |  | Functional  Classes | Common to Q- and R-type |
| Other | SepL(ECs4557), |  | Transcriptional  Regulator | Cell division (*yjdB, cs2214*), Cro (ECs0275), *srlR*, *yfjR*, *ymcg*, *yehl*, ECs2925 and ECs3032, |  | Transcriptional Regulator | DicB, LrhA, ECs4349 (GntR family) |
| Enzymes | N5-glutamine S-adenosyl-L-methionine-dependent methyltransferase (yfcB), NADH dehydrogenase (ndh), bifunctional aspartate kinase II/ homoserine dehydrogenase II (metL), geranyl transferase (ipsA), integrase (ECs0289),  hypothetical proteins: ECs0606, ECs1070, ECs2229, ECs2749, ECs2755, ECs3216, |  | Enzymes | GDP/GTP pyrophospho  kinase ***relA***, nitrate reductase (napA), urease (UreG), phosphoglycerate mutase (ECs4490), ATP-dependent protease La (ECs0493),  endopeptidase ECs2739, excisionase ECs0801, fructose-bisphosphate aldolase, glutamate synthase subunit beta(gltD), glycosyl transferases (ECs2843 and ECs2845), hydrogenase 2 maturation endopeptidase (hybD), mannose-1P guanyl-transferase (*manC*), homocytein S-methyl transferase (*metE*), pyruvate dehydrogenase (poxB), amino-transferase (ybdl), short-chain acyl CoA synthetase (ydiD) |  | Enzymes | ATP-dependent protease ECs4859, PTS system galactitol-specific enzyme IIC(ECs2895), SepL (ECs4557), bifunctional aspartate kinase II/homoserine dehydrogenase II, endonuclease V, geranyltranstransferase(ECs0475), integrase (ECs0289), phospho-2-dehydro-3-deoxyheptonate aldolase,  host specificity protein (ECs1990), hypothetical proteins:  ECs0606, ECs1070, ECs1580, ECs2229, ECs2749, ECs2755, ECs3216, |
|  |  |  | Transporters | *narU*  (NO_3_^-^/ NO_2_^-^ transporter), (ECs2641 and ECs3071 (permeases) |  |  |  |
|  |  |  | Phage-related functions | phage replication protein, tail length tape measure protein, tail protein(ECs2643 and ECs4984), terminase large subunit (ECs1792) |  |  |  |
|  |  |  | Chaperone | bfpT-regulated chaperone-like protein |  |  |  |
|  |  |  | Adhesion/  pathogenicity | Blue light, low temperature and stress induced protein (ymgC), EspF-like protein, FidL-like protein, invasin (ECs5290), complement resistance protein (ECs1312), curli assembly protein CsgE, fimbrial protein (ECs4427), flagellin structural protein (ECs0424) |  |  |  |
