# Supplementary material for: Influence of Glucose Availability and CRP Acetylation on the Genome-Wide Transcriptional Response of Escherichia coli: Assessment by an Optimized Factorial Microarray Analysis
Source: Front Microbiol. 2018 May 23;9:941. doi: 10.3389/fmicb.2018.00941 (PMC5974110; doi:10.3389/fmicb.2018.00941)
Supplement: Table S16 — Ontology analysis for the Δcrp(Q-type) and Δcrp(R-type) mutants in the case of up-regulated genes during the stationary phase. [file Supplementary_TABLE_S16.docx]

| **Influence of glucose availability and CRP acetylation on the genome-wide transcriptional response of *Escherichia coli*: assessment by an optimized factorial microarray analysis**  Daniel V. Guebel^1^ and Néstor V. Torres^2*^  ^1^Biotechnology Counselling Services. Buenos Aires. Argentina; ^2^Systems Biology and Mathematical Modelling Group. Department of Biochemistry, Microbiology, Cellular Biology and Genetics. Institute of Biomedical Technologies. Center for Biomedical Research of the Canary Islands. University of La Laguna. San Cristóbal de la Laguna. Spain.  *e-mail (NVT): [ntorres@ull.edu.es](mailto:ntorres@ull.edu.es) |
| --- |

**Table S16 | Main functional classes present in the up-regulated genes during the stationary-growth phase according to the acetylation stage of CRP**

| CRP-dependence (up-regulated genes) | | | | |
| --- | --- | --- | --- | --- |
| Functional  Classes | Exclusive on Q-Type |  | Functional  Classes | Exclusive on R-Type |
| Enzymes | Arginine and Proline catabolism **(FDR=1.8 x 10^-6^):**  arginine succinyltransferase, bifunctionals, succinylornithine transaminase/ acetylornithine transaminase (argD), succinylarginine dihydrolase (ECs2451), succinylglutamate desuccinylase (ECs2450), succinylglutamic semialdehyde dehydrogenase (astD)  Other Enzymes:  hydrogenase 1 maturation protease, hydrogenase-1 operon protein HyaE , hydrogenase 1 b-type cytochrome subunit, hydrogenase 1 large subunit (ECs1129), hydrogenase-1 small subunit (ECs1128), hydrogenase-1 operon protein HyaF (involved in nickel incorporation into hydrogenase-1 proteins). |  | Enzymes | 4-aminobutyrate aminotransferase, DNA mismatch repair protein(mut), L-arabinose isomerase (ECs0066), NAD(P)H-dependent FMN reductase, NADH dehydrogenase I subunit F, anthranilate synthase component I, bifunctional diaminohydroxyphosphoribosylamino pyrimidine deaminase/5-amino-6-(5-phosphoribosylamino)uracil reductase (ribD), carboxylesterase BioH, deoxyribonucleotide triphosphate pyrophosphatase, dihydroorotate dehydrogenase 2, fucose synthetase, nicotinate-nucleotide--dimethylbenzimidazole phosphoribosyltransferase (cobT), oxidoreductase (ECs1878), phospho-2-dehydro-3-deoxyheptonate aldolase, phosphoribosylamine--glycine ligase, phosphotransferase system enzyme IIA (ECs4350), pyruvate dehydrogenase, rhamnulose-1-phosphate aldolase (rhaD), siroheme synthase (cysG), threonyl-tRNA synthetase (ECs2426) |
| Transporters | L-arabinose ABC transporter permease (araH),  L-arabinose transporter ATP-binding protein (araG),  L-arabinose-binding periplasmic protein (ECs2609),  glucitol/sorbitol-specific enzyme IIC component of PTS(srlA),  ABC transporter ATP-binding protein ytfR (ECs5206),  sugar transport system permease ytfT |  | Transporters | galactose-proton symport (ECs3819), sorbose-permease PTS system IIC component (ECs5000), PEP-protein phosphotransferase system enzyme I (ECs4877),  ATP-binding protein livG, hemin permease (ECs4386), histidine/lysine/arginine/ornithine transporter (ECs3190), leucine/isoleucine/valine permease livM, peptide ABC transporter ATP-binding protein (dppD), peptide ABC transporter (ECs4422), periplasmic binding protein of high-affinity leucine-specific transport system (ECs4305), phosphonate/organophosphate ester transporter subunit (ECs5088), ECs4272, ECs2476,ECs0740, permease ECs3056, YdeE (ECs2141), arginine exporter protein (ECs3794), multidrug efflux system protein MdtO (ECs5063), peptide ABC transporter (ECs4422), phenylalanine transporter (ECs0614), sorbose-permease PTS system IIC component (ECs5000) |
|  |  |  | Two-component System | histidine protein kinase (ECs5074), hybrid sensory histidine kinase TorS (ECs1148), UhpA family transcriptional regulator (ECs4606), potassium-transporting ATPase subunit A (ECs0726), cryptic nitrate reductase 2 subunit alpha (ECs2071), ECs4272 |
|  |  |  | Outer-membrane | ECs1122, ECs1236, ECs2915 |
| Transcriptional regulator | LACI-type transcriptional regulator ytfQ |  | Tail Assembly | ECs0841, ECs1987 |
| ncRNA | rybB |  | Other | Acyltransferase (ECs3232), holin (ECs1100, ECs1212, ECs1782), hypothetical proteins (ECs0224, ECs2748, ECs4591)  beta-lactamase(ampC) |
|  |  |  | Bacterial secretion system | Lipoprotein (ECs0224), hypothetical proteins (ECs0224, ECs0234, ECs0607) |
|  |  |  | Siderophore biosynthesis (non-ribosomal peptides) | entE, entF |
