# Supplementary material for: Influence of Glucose Availability and CRP Acetylation on the Genome-Wide Transcriptional Response of Escherichia coli: Assessment by an Optimized Factorial Microarray Analysis
Source: Front Microbiol. 2018 May 23;9:941. doi: 10.3389/fmicb.2018.00941 (PMC5974110; doi:10.3389/fmicb.2018.00941)
Supplement: Table S17 — Ontology analysis for the Δcrp(Q-type) mutant in the case of down-regulated genes during the stationary phase. [file Supplementary_TABLE_S17.docx]

| **Influence of glucose availability and CRP acetylation on the genome-wide transcriptional response of *Escherichia coli*: assessment by an optimized factorial microarray analysis**  Daniel V. Guebel^1^ and Néstor V. Torres^2*^  ^1^Biotechnology Counselling Services. Buenos Aires. Argentina; ^2^Systems Biology and Mathematical Modelling Group. Department of Biochemistry, Microbiology, Cellular Biology and Genetics. Institute of Biomedical Technologies. Center for Biomedical Research of the Canary Islands. University of La Laguna. San Cristóbal de la Laguna. Spain.  *e-mail (NVT): [ntorres@ull.edu.es](mailto:ntorres@ull.edu.es) |
| --- |

**Table S17 | Main functional classes present in the down-regulated genes during the stationary-growth phase as depending on CRP^acetylated^**

| CRP-dependence (down-regulated genes) | |
| --- | --- |
| Functional  Classes | Exclusive on Q-Type |
| Ribosomal proteins | rpsJ, rpsP, rpsS, rpsT, rpsF, rplB,rplC, rplD, rplM, rpmC,  50S ribosomal protein L25 (ECs3077) **(FDR=1.2x10^-8^)** |
| Pyrimidine metabolism | aspartate carbamoyltransferase (ECs5221), aspartate carbamoyltransferase (pyrB),  carbamoyl phosphate synthase large subunit (carB), carbamoyl phosphate synthase small subunit (ECs0035), cytosine deaminas (ECs0390), dihydro-orotate oxidase, FMN-linked (pyrD), dihydroorotase (ECs1440), uracil phosphoribosyltransferase (upp), 5-(carboxyamino)imidazole ribonucleotide synthase (ECs0584) **(FDR= 4.9x10^-5^)** |
| ATP synthesis coupled to H^+^ transport | ATP synthase F0F1 subunit A (ECs4680),  ATP synthase F0F1 subunit B (ECs4678),  ATP synthase F0F1 subunit C (ECs4679,)  ATP synthase F0F1 subunit beta (ECs4674),  ATP synthase F0F1 subunit delta (ECs4677),  cytochrome o ubiquinol oxidase subunit I (ECs0485),  cytochrome o ubiquinol oxidase subunit IV (ECs0483) **(FDR=1.1 x 10^-4^)** |
| Aminoacid metabolism | Cysteine and Methionine metabolism: 5-methyltetrahydropteroyltriglutamate/homocysteine S-methyltransferase (ECs4759), cysteine synthase B (cysM), methionine adenosyltransferase(ECs3818); Glycine, Serine and Threonine: D-3-phosphoglycerate dehydrogenase (ECs3784), bifunctional aspartokinase I/homoserine dehydrogenase I (thrA), serine hydroxymethyltransferase (glyA); Alanine, Aspartate and Glutamate metabolism: glutamate dehydrogenase (ECs2467), glutamine synthetase (glnA), glutamate synthase, 4Fe-4S protein, small subunit (gltD), glutamate synthase, large subunit (gltB); Phenylalanine, Tyrosine and Tryptophane metabolism: phospho-2-dehydro-3-deoxyheptonate aldolase (ECs0782) |
| Transporters | DL-methionine transporter substrate-binding subunit (metQ), arginine ABC transporter periplasmic binding protein (artJ), branched-chain amino acid ABC transporter ATPase (livF), branched-chain amino acid ABC transporter ATPase (livG); periplasmic binding protein of high-affinity amino acid transport system(ECs4309) |
| Other enzymes | aconitate hydratase B (ECs0122), sulfate adenylyltransferase, subunit 2 (cysD),  sulfite reductase subunit alpha (cysJ) |
| Flagellar assembly | flagellar basal-body rod protein FlgB, flagellar basal body rod protein FlgC, flagellar basal body rod modification protein(flgD), flagellar biosynthesis sigma factor (fliA), flagellar component of cell-proximal portion of basal-body rod(flgF), flagellar filament capping protein(fliD), flagellar hook protein FlgE, |
| Two-component system | outer membrane protein F(ECs1012), purine-binding chemotaxis protein (ECs2597), flagellar biosynthesis sigma factor (fliA), glutamine synthetase (glnA) |
